# Effects of an Endophytic Fungus *Umbelopsis dimorpha* on the Secondary Metabolites of Host–Plant *Kadsura angustifolia*

**DOI:** 10.3389/fmicb.2018.02845

**Published:** 2018-11-22

**Authors:** Dan Qin, Ling Wang, Meijun Han, Junqi Wang, Hongchuan Song, Xiao Yan, Xiaoxiang Duan, Jinyan Dong

**Affiliations:** ^1^Key Laboratory of Eco-Environments in Three Gorges Reservoir Region (Ministry of Education), School of Life Sciences, Southwest University, Chongqing, China; ^2^Solar Energy Research Institute, School of Energy and Environment Science, Yunnan Normal University, Kunming, China

**Keywords:** *Kadsura angustifolia*, *Umbelopsis dimorpha*, highly oxygenated schitriterpenoids/schinortriterpenoids, microbial fermentation, fungus–plant interaction

## Abstract

Fungal endophytes live widely inside plant tissues and some have been revealed to provide benefits to their host and ecological environment. Considering the fact that endophytes are engaged in remarkably stable long-term interactions with the host for their whole life cycle, it’s conceivable that both partners have substantial influence on each other’s metabolic processes. Here, we investigated the fermented products of an endophytic fungus *Umbelopsis dimorpha* SWUKD3.1410 grown on host–plant *Kadsura angustifolia* and wheat bran, respectively, to assess the impact of SWUKD3.1410 on the secondary metabolites of *K. angustifolia*. Twenty compounds (**1**–**20**) were isolated and identified as 11 schitriterpenoids (**1**–**9**, **17**–**18**), two lignans (**10**, **20**), two sesquiterpenoids (**11**–**12**), one trinorsesquiterpenoid (**13**), one monoterpene (**14**), one sterol (**19**), and two simple aromatic compounds (**15**–**16**) by the extensive 1D-, 2D-NMR and HR-ESI-MS data analysis. Except for nigranoic acid (**1**), compounds **2**–**19** have been firstly found from *K. angustifolia*. Of them, metabolites **2**, **11**, and **14** were identified to be new. Obtained results indicated that *U. dimorpha* SWUKD3.1410 could not only produce the same/similar components as its host does, and modify the host–plant components, but also enhance the production of these highly oxygenated schitriterpenoids/schinortriterpenoids in plants. This study suggested an interesting prospective for setting up alternative processing techniques to improve the quality of crude drugs derived from *K. angustifolia* and increase their values.

## Introduction

Endophytic fungi or endophytes are diverse polyphyletic groups of microorganisms that colonize intracellularly or intercellularly the living tissues of host plant without triggering any visible external sign of infection (Tan and Zou, 2001; Kaul et al., 2012). Some co-existing endophytic fungi are considered to be plant mutualists: they receive nutrition and protection from the host plant to proliferate while the host plant may benefit from enhanced competitive abilities and increased tolerances to pathogens, herbivores, and various abiotic stresses (Schulz and Boyle, 2005; Kusari et al., 2012). Undoubtedly, the key to understanding the endophytic functions is to elucidate the mechanisms involved in plant–endophyte interactions (Herre et al., 2007). Nevertheless, the mechanisms of fungus–plant interaction have not been fully discerned owing to the complexity and diversity of the partners related (Rodriguez et al., 2009; de Siqueira et al., 2017). It is essential to employ simplified systems in investigating interactions of fungi and plant hosts (Schulz and Boyle, 2005). The productions of secondary metabolites, an interesting aspect of the fungus–plant interactions, have been demonstrated to play a critical role in their interactions, especially for defense, signaling purposes, and regulation of the symbiosis (Schulz and Boyle, 2005). Some reports have demonstrated that endophytic fungi can significantly affect the formation of metabolic products in plants, such as inducing host metabolism and transforming host components, and *vice versa* (Qawasmeh et al., 2012; Tian et al., 2014; Ludwig-Müller, 2015). Considering the fact that endophytes are continuously interacting with their hosts, it’s logical to suggest that both partners have a vital impact on each other’s metabolic processes (Tian et al., 2014).

It has been proposed that endophytic fungi have developed versatile biosynthetic capabilities (including synthesis, transformation, and degradation of host plant metabolites) through the long period of coevolution and genetic recombination with their hosts (Borges et al., 2009). These special symbiotic relationships with the host plant forced the endophytic fungi to dispose several toxic compounds produced by their hosts as defense against other organisms. The existence of biodegradation and biotransformation processes of the toxic substances by the help of certain specific enzymes has enabled them to survive (Borges et al., 2009). Werner et al. (1997) revealed that several endophytes from the roots of *Aphelandra* plant could well-metabolize aphelandrine, a macrocylic polyamine alkaloid isolated in the roots of different species of the genus *Aphelandra*, which was the first report on the biotransformation of endophytic fungi. After that, similar findings that endophytic fungi were capable of transforming some natural products and metabolizing drugs have been reported continuously (Gniłka et al., 2015; Smith et al., 2015). Specifically, compared to traditional method, the microbial transformation of raw herb powder has been reported to produce a higher yield of active components (Liu et al., 2010; Zhu et al., 2010; Ding et al., 2014). It has also been found that endophytic fungi may be adept in modifying chemical structures with a high degree of stereo-specificity (Borges et al., 2009). Some reactions are very similar to mammalian phase I metabolism (Borges et al., 2009). Therefore, more recently, endophytes have gained attention as potential sources of novel biocatalysts in the chemical transformation of natural products and drugs (Pimentel et al., 2010).

**FIGURE 1:**
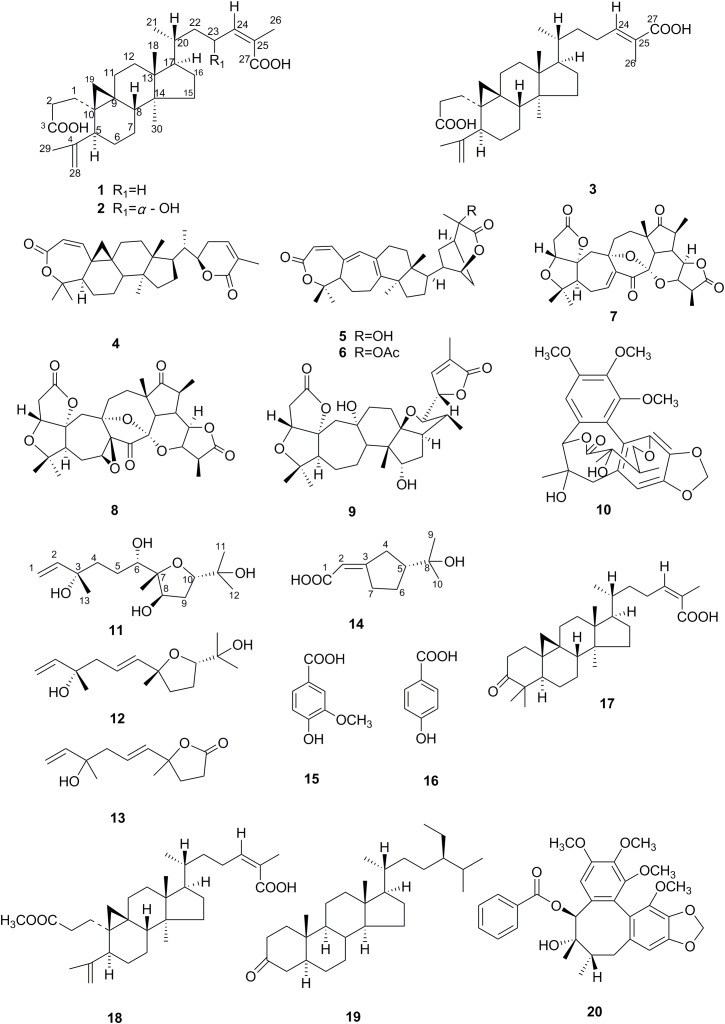
Structures of compounds **1**–**20**.

The family Schisandraceae contains the two genera, *Schisandra* and *Kadsura.* There are about 50 species in total belonging to this family, which are mainly distributed in Southeast Asia and North America (Xiao et al., 2010b). Many species of this familya have been used for generations in China as folk medicine to treat premature ejaculation, hepatitis, chronic dysentery, cough, and insomnia (Xiao et al., 2010b; Liang et al., 2012). Since the first discovery of the 3,4-seco-cycloartene nigranoic acid from *S. nigra* in 1972 (Sun et al., 1996), Schisandraceae plants have been a hot topic within the medicinal chemistry and drug discovery communities because a plethora of schtriterpenoids/schnortriterpenoids with 47 skeletons has been reported over the years with far more than 1000 terpene-type compounds been estimated to be produced by the plants of Schisandraceae family (Xiao et al., 2008; Xia et al., 2014; Shi et al., 2015; Zhou et al., 2016). These fascinating molecules have been reported to possess various beneficial bioactivities such as antihepatitis, antitumor and anti-HIV, and have attracted wide attention of phytochemists and pharmacologists (Xiao et al., 2008). As expected, the chemical synthesis of these specialized metabolites is quite difficult and commercially unfeasible owing to the complicated stereo-chemical rings with multiple chiral centers. Structurally, they are generally supposed to derive from the common cycloartane skeleton by ring rearrangement, oxidative cleavage, loss of carbons, and other reactions (Xia et al., 2014; Shi et al., 2015). However, many attempts to obtain the highly oxygenated triterpenoids failed in previous microbial biotransformation made directly on the substrates of cycloartane-type triterpenes, such as nigranoic acid (Dong et al., 2007a,b,Sun et al., 2013) and cycloartenol (Kuban et al., 2010, 2013). Recently, we noticed a report that cultivation of endophytes without their host plant might result in the loss of the desired compound synthesis (Ludwig-Müller, 2015). Accordingly, we assumed that partial or complete biosynthesis pathways for highly oxygenated schitriterpenoids/schinortriterpenoids can be attributed to the host environment, which may trigger the expression of silent biosynthetic pathways, or result in a change in expression of the enzymes related to the cyclization and oxidation of squalene. Our previous report indicated that *Kadsura angustifolia* (Lem.) Smith could be a community model for studies of their metabolic relationships between the endophytic fungi and host plant due to its relative single chemical composition, in which the isolated yield of nigranoic acid in the root and stem of *K. angustifolia* collected at Xichou of Yunnan province can reach up to 3.8% of the dry weight of the separate origin (Sun et al., 2011; Huang et al., 2015). More than 426 isolates were identified by morphological and molecular analysis (Huang et al., 2015). Among them, *Umbelopsis dimorpha* SWUKD3.1410 appeared to be relatively specific to *K. angustifolia* and showed remarkable biotransformative activities (Huang et al., 2015). Thus, as reported by Tian et al. (2014), in order to evaluate the impact of endophytic fungal inoculants on the production of secondary metabolites of host–plant *K. angustifolia*, the strain SWUKD3.1410 was selected to grow on different substrates. Furthermore, the fermentative cultures of *K. angustifolia* and wheat bran were chemically investigated, which led to the isolation and structural elucidation of 20 compounds (**1**–**20**) (Figure 1), including 11 schitriterpenoids (**1**–**9**, **17**–**18**). Among them, metabolites **2**–**20** have never been found before from *K. angustifolia*, and compounds **2**, **11**, and **14** were identified to be new. Taken together with the reported components of *K. angustifolia* (Chen et al., 1998; Gao et al., 2008; Sun et al., 2011) and wheat bran (Posner, 2009; Zhu et al., 2013, 2015; Prinsen et al., 2014; Geng et al., 2015), we found the *U. dimorpha* SWUKD3.1410 cannot only yield the same and similar compounds as those present in its host–plant *K. angustifolia*, but also can significantly modify the host plant components and enhance the production of those highly oxygenated schitriterpenoids/schinortriterpenoids. This work will provide new sight for researchers to make better use of the beneficial symbiosis and expand the way for obtaining bioactive natural products from certain medicinal plants.

## Materials and Methods

### General Experimental Procedures

NMR spectrums (^1^H-NMR, ^13^C-NMR, DEPT, HMQC, HMBC, ^1^H-^1^HCOSY, NOESY) were recorded on a Bruker Avance 600 spectrometer (Fällanden, Bruker BioSpin, Switzerland). The chemical shifts (δ) were reported in ppm, and the coupling constants in hertz (Hz). Standard pulse sequences were used for DEPT, NOESY, HMQC, and HMBC experiments. Ultraviolet (UV) spectra were measured with a Shimadzu double-beam 210A spectrophometer (Shimadzu). Optical rotations were measured on a Jasco DIP-370 polarimeter (JASCO Corporation, Japan). Infrared (IR) spectra were obtained in KBr pellets with a Bio-Rad FTS-135 spectrophotometer (Bio-Rad). MSs were taken on a VG Auto Spec-3000 mass spectrometer (VG Instruments, East Sussex, England). HR-MSs (ESI-TOF) data were recorded with an API QSTAR Pulsar 1 spectrometer.

### Chemicals

All solvents used for product isolation were of analytical grade or higher. Silica gel (200–300 mesh, Qingdao Marine Chemical Factory, Qingdao, China) and Sephadex LH-20 (Amersham Pharmacia, Uppsala, Sweden) were used for column chromatography. Pre-coated silica gel GF254 plates (Qingdao Marine Chemical Factory, Qingdao) were used for TLC. Fractions were monitored by TLC, and spots were visualized by heating silica gel plates sprayed with 5% H_2_SO_4_ in ethanol.

### Strains and Morphology

The endophytic fungus SWUKD3.1410 used in this study was isolated from fresh, healthy branches of *K. angustifolia* collected in November (dry season) 2012 at Xichou (N23°26′18.10″, E104°40′19.92″) of Yunnan province of China, lying approximately 55.8 km apart (Huang et al., 2015). The strain was preliminarily identified based on morphological characterization after 7 days in a PDA (potato 200 g, glucose 20 g, agar 18 g, and water 1000 mL) medium. To stimulate sporulation, the strain was cultured in the water-agar plates and placed in darkness at 28°C for up to 20 days. For morphological studies, slide cultures were prepared and stained with lactophenol cotton blue dye. Using a Canon EOS 5D Mark II digital camera, morphology of colonies (texture, color, and presence of pigmentation) grown on PDA media was photographed as described in Simoes et al. (2013). The microscopic characteristics (morphology of vegetative spores’ structures) were observed with an Axio Lab. A1 microscope (Carl Zeiss, Germany) equipped with an Axiocam ICc 5 digital camera (Carl Zeiss Vision, Germany). Standard taxonomic manuals were used to identify the fungal genus and species (Mahoney et al., 2004; Wang et al., 2013).

### DNA Extraction, PCR Amplification, Sequencing, and Phylogenetic Analysis

The endophytic fungus SWUKD3.1410 was inoculated onto potato dextrose broth (PDB) and cultured at 28°C for 3 days. Then, the rejuvenated fungus was re-inoculated into 250 mL Erlenmeyer flasks containing 70 mL broth and incubated at 180 rpm at 28°C for 7 days. Finally, the culture was harvested by centrifugation at 12,000 rpm for 10 min. Genomic DNA was extracted from 0.5 to 1 g chilled mycelia in liquid nitrogen using the CTAB method (Zhang et al., 2006). The fungal ITS fragments (ITS1-5.8S-ITS2 rDNA) were amplified using the universal primers ITS1 and ITS4 (White et al., 1990). The PCR reaction mixtures (25 μL) contained 1 μl of genomic DNA, 2.5 μl of PCR buffer, 2 μl of MgCl_2_, 2 μl of forward and reverse primers, 2 μl of deoxyribonucleotide triphosphate (dNTP), 0.25 μl of rTaq polymerase (TaKaRa Biotechnology, Ltd., China) and 13.25 μl of double distilled water. The PCR reaction was composed of an initial denaturing step at 94°C for 3 min, followed by 32 amplification cycles at 94°C for 30 s, 56°C for 30 s, 72°C for 90 s, and a final extension at 72°C for 10 min. The PCR products were analyzed by agarose gel electrophoresis and purified using a DNA gel extraction kit (Omega Biotechnology, Ltd., China). The purified PCR product was sequenced using the same primers (BGI-Beijing, Beijing, China).

The endophytic fungi were classified by comparing the ITS sequences of endophytic fungi with the data available in NCBI using BLAST search. The resulting sequences were aligned with the Clustal X software (Larkin et al., 2007), with gaps treated as missing data. Phylogenetic tree was built by the neighbor-joining method using Mega6.0 software. The bootstrap was 1000 replications to assess the reliability of the inferred tree (Tamura et al., 2011).

### Plant Material and Solid-State Fermentation of the Fungal Strain SWUKD3.1410

The stems and roots of *K. angustifolia* were collected in Xichou, Yunnan, China, in October 2014. The sample was identified by Prof. H. P. Deng (Southwest University), and a voucher specimen has been deposited in the herbarium of Southwest University. Fresh *K. angustifolia* plant material was cut to a size of 1 cm and air-dried.

In order to assess whether the high temperature sterilization process will affect the chemical components of *K. angustifolia* plant material and wheat bran, 6 g wet *K. angustifolia* plant material and wheat bran were put into 50 mL Erlenmeyer flasks respectively, and then they were routinely sterilized in autoclave (121°C, 30 min). After sterilization was finished, each flask was directly dried in oven at 55°C for 48 h, and then 30 ml acetone was added into the flask. The dried extracts were soaked at room temperature for 4 days, followed by evaporating all the extracts in a rotary evaporator under reduced pressure. Acetone extracts of 6 g non-sterilized dry *K. angustifolia* plant material and wheat bran were served as controls respectively. The process was similar as above mentioned. And the corresponding extracts were used for further TLC and HPLC analysis.

*Umbelopsis dimorpha* SWUKD3.1410 was activated on the surface of PDA at 28°C for 5 days and the mycelium-containing agar was then cut into 5-mm disks and inoculated in 50 mL Erlenmeyer flasks, containing 6 g of sterilized wet solid medium (a mixture of the stem and root segments of *K. angustifolia*) (6 g), which was adjusted to 60–70% (w/v) by distilled water containing 1% (w/v) CaCO_3_. The cultivation was performed in darkness at 28°C without shaking for 25 days. Meanwhile, the other three different medium, including wheat bran (6 g), the leached residue of *K. angustifolia* (*K. angustifolia* plant materials which were extracted with acetone) (6 g), as well as a mixture of wheat bran (2 g) and the stem and root segments of *K. angustifolia* (4 g), were used to culture *U. dimorpha* SWUKD3.1410 under the same conditions, respectively. After fermentation was finished, each flask was directly dried in oven at 55°C for 48 h, and then 30 ml acetone was added into the flask. The dried extracts were soaked at room temperature for 4 days, followed by evaporating all the extracts in a rotary evaporator under reduced pressure. And the corresponding residues left were used for further TLC and HPLC analysis. Substrate consisting of pure nigranoic acid, acetone extracts of sterilized *K. angustifolia* plant materials and wheat bran served as control, respectively.

The preparative-scale biotransformation and fermentation of the fungus was performed in 500 mL Erlenmeyer flasks containing wet *K. angustifolia* (100 g) or wet wheat bran (100 g). The process was similar as above mentioned. In brief, 20 5-mm disks were inoculated into the sterilized solid medium, and fermented in darkness at 28°C without shaking for 50 days. The fermented materials including all the mycelia and solid medium were collected together and dried at 60°C. All dried materials were extracted with acetone for 4 days for three times until no spots in the extracts were detected by TLC analysis. All the extracts were then put together and evaporated under reduced pressure in a rotary evaporator. After removing the solvent, 110 and 60 g dark brown residues were obtained from fermentation cultures of 5 kg *K. angustifolia* plant materials and 5 kg bran, respectively.

### TLC and HPLC Analysis of Fermented Samples

The acetone extract of each fermented sample in preliminary experiments (10 μg/spot), was spotted on the start line of a 0.5 cm (5 cm × 10 cm) silica gel GF254 fluorescent TLC plates and developed in a solvent system (chloroform/ethyl acetate/glacial acetic acid at 6:4:0.1 v/v/v). In addition, acetone solutions of pure nigranoic acid and non-fermented *K. angustifolia* plant materials were also used as controls in TLC analysis. After air-dry, the plates were examined with a hand-held UV lamp at 254 nm. Then, the plates were sprayed with a chromogenic agent, concentrated sulfuric acid/absolute ethanol (5:95 v/v), and heated for 5 min at 150°C.

Additionally, each methanol solution of the above-mentioned fermented sample extracts (5 μl of a 1:100 enriched extract), as well as methanol solutions of pure nigranoic acid (5 μl of 0.1 mg/ml), sterilized *K. angustifolia* plant materials and sterilized wheat bran control (5 μl of a 1:100 enriched extract), were further analyzed on an Agilent 1100 series HPLC equipped with a Agilent ZORBAX Eclipse XDB-C18 column (4.6 mm × 250 mm, 5 μm). The mobile phase consisted of acetonitrile and methanol with 0.05% (vol/vol) phosphoric acid at a constant flow rate of 1 ml/min. The initial mobile phase consisted of MeCN-H_2_O at 20:80% (v/v), and this was maintained for 10 min, after which the gradient was change to MeCN-0.05% phosphoric acid at 45:55% (v/v) over a period of 10 min. This gradient was then returned to 85:15% (v/v) for 10 min, and then held for 10 min postrun reconditioning. The column temperature was held constant at 30°C, and the metabolites were monitored using a detection wavelength of 217 nm.

### Extraction and Purification of Secondary Metabolites of Endophytic Fungus SWUKD3.1410 Grown on *K. angustifolia*

The residue (about 110 g) was subjected to dry column on silica gel (200–300 mesh, 4.0 cm × 90 cm, with 400 g of silica gel), and eluted with gradient mixtures of petroleum ether-AcOEt (1:0-0:1) to afford five main fractions (Frs. A-E). Fraction A (3.2 g) was further separated on Sephadex LH-20 column eluted with MeOH to obtain 51 fractions (Frs. A1-55). The combined Frs. A21-27 (400 mg) was further purified by a silica gel column chromatography (200–300 mesh, 1.2 cm × 32 cm, 16 g silica gel), and eluted with gradient mixtures of CHCl_3_-AcOEt (9:1-8:2) to yield compounds **10** (13 mg) and **3** (9 mg). The combined Frs. A29-37 (260 mg) was further supplied to a column chromatography on silica gel (200–300 mesh, 1.2 cm × 32 cm, 12 g silica gel), and eluted with gradient mixtures of petroleum ether-AcOEt (7:3-6:4) to give compound **14** (4 mg). The combined Frs. A40–51 (90 mg) was purified by Sephadex LH-20 and further separated by preparative TLC to give compounds **16** (2 mg) and **15** (3 mg). Fraction B (2.1 g) was further purified to Sephadex LH-20 column eluted with acetone to afford 62 fractions (Frs. B1-62). The combined Frs. B16-23 (200 mg) was further chromatographed on silica gel column (200–300 mesh, 1.2 cm × 32 cm, with 12 g of silica gel), and eluted with gradient mixtures of CHCl_3_-AcOEt (6:4-5:5) to give compounds **1** (50 mg), **5** (17 mg), **7** (3 mg), and **6** (21 mg). In the same manner, Fr. C (800 mg) was purified to give compounds **1** (25 mg), **8** (2.7 mg), and **12** (15 mg). Fraction D (3.1 g) was chromatographed on Sephadex LH-20 column eluted with MeOH to afforded 78 fractions (Frs. D1-67). The combined Frs. D8-31 (650 mg) were repeatedly separated by a silica gel column chromatography (200–300 mesh, 1.7 cm × 38 cm, 30 g silica gel), and eluted with gradient mixtures of CHCl_3_-AcOEt (5:5-3:7) to yield compounds **1** (65 mg), **2** (8 mg), **9** (2 mg), and **13** (7 mg). Fraction E (2.8 g) were further separated by a silica gel column chromatography (200–300 mesh, 2.6 cm × 38 cm, 80 g silica gel), and eluted with mixtures of CHCl_3_- AcOEt (3:7) to yield compounds **4** (13 mg) and **11** (3 mg).

### Extraction and Purification of Secondary Metabolites of Fungus SWUKD3.1410 Grown on Wheat Bran

The residue (about 60 g) was subjected to dry column on silica gel (200–300 mesh, 4.0 cm × 90 cm, with 400 g of silica gel), and eluted with gradient mixtures of petroleum ether-AcOEt (1:0-0:1), to afford six fractions (Frs. A-F). Fraction B (2 g) was further supplied to a column chromatography on silica gel (200–300 mesh, 2.0 cm × 40 cm, with 53 g of silica gel), and eluted with gradient mixtures of petroleum ether-AcOEt (8:2-6:4) to give compounds **17** (25 mg) and **19** (4 mg). Fraction C (1.7 g) were chromatographed on silica gel (200–300 mesh, 2.0 cm × 40 cm, with 53 g of silica gel), and eluted with gradient mixtures of petroleum ether-AcOEt (6:4-5:5) to give compounds **18** (7 mg) and **1** (80 mg). Fraction D (1.1 g) was further supplied to a Sephadex LH-20 column eluted with methanol to afford compounds **20** (5.3 mg) and **4** (5 mg).

### Structural Determination of the New Secondary Metabolites

23α-hydroxynigranoic acid (**2**): acicular crystal: : +15.3° (c 0.18, MeOH); UV (MeOH) λ_max (logε): 205.00 (4.20), 403.00 (2.48) and 549.5 (2.27) nm; IR (KBr): ν_max_ = 3425, 3068, 3038, 2928, 2872, 1695, 1570, 1451, 1404, 1303, 1283, 1252, 1155, 1125, 1055, 979, 891, 858, 826, 777, 720, 688, 606, 570, 544, 479, 454, 430 cm^-1^; ^1^H and ^13^C-NMR (CD_3_OD, 600 MHz) data (Table 1); HR-ESI-MS *m/z*: 509.3228 ([M+Na]^+^, calc. for C_30_H_46_O_5_Na, 509.3237).

**Table 1:** The ^1^H- and ^13^C-NMR spectral data of compounds **2**, **11**, and **14** (600 MHz, *J* in Hz).

No.	2^a^		11^b^		14^c^	
	δ_H_	δ_C_	δ_H_	δ_C_	δ_H_	δ_C_
1	1.92(m); 1.22(m)	28.1t	4.95(dd,1.6,10.8); 5.19(dd,1.6,17.3)	111.3t		170.1s
2	2.38(m); 2.11(m)	32.2t	5.90(dd,10.8)	147.2d	7.04(s)	140.5d
3		175.3s		72.9s		129.9s
3-O**H**			3.78(s)			
4		149.6s	1.52(m); 1.79(m)	40.7t	2.03(m); 2.34(m)	26.5t
5	2.43(m)	45.3d	1.44(m); 1.63(m)	26.9t	1.54(m)	44.1d
6	1.45(m); 1.06(m)	27.3t	3.38(d,10.2)	78.6d	1.98(m); 1.24(m)	23.3t
6-O**H**			4.27(s)			
7	1,24(m); 1.06(m)	25.1t		88.7s	2.17(m); 2.52(m)	25.0t
8	1.53(m)	47.7d	4.32(m)	73.2d		72.3s
8-O**H**			3.74(d,4.7)			
9		21.3s	1.79(m); 2.24(m)	36.7t	1.21(s)	27.2q
10		29.5s	3.88 (t,7.6,15.1)	83.2d	1.22(s)	27.4q
11	2.06(m); 120(m)	26.8t		71.4s		
11-O**H**			3.57(s)			
12	1.61(m); 1.57(m)	33.2t	1.18(s)	27.4q		
13		45.4s	1.05(s)	26.5q		
14		50.9s	1.09(s)	17.4q		
15	1.36(m); 1.26(m)	35.7t	1.21(s)	28.6q		
16	1.83(m); 1.26(m)	27.7t				
17	1.54(m)	52.7d				
18	0.96(s)	18.4q				
19	0.69(d,3.8); 0.38(d,3.8)	29.9t				
20	1.68(m)	32.1d				
21	0.92(d,6.2)	18.4q				
22	1.55(m); 0.89(m)	43.4t				
23	4.33(m)	64.9d				
24	6.53(d,7.9)	145.3d				
25		126.7s				
26	1.71(s)	13.0q				
27		169.8s				
28	4.82(m); 4.71(m)	111.9t				
29	1.66(s)	20.2q				
30	0.90(s)	19.5q				


*^a^NMR in CD3OD, ^*b*^NMR in CD3COCD3,^*c*^NMR in CDCl3.*

6,8-Dihydroxy-schensianol A (**11**): colorless oil: : +22.2° (c 0.12, MeOH); UV (MeOH) λ_max (logε): 202.00 (3.79), 251.60 (2.51) and 298.40 (1.77) nm; IR (KBr): ν_max_ = 3406, 3396, 3387, 3088, 2974, 2933, 2876, 2641, 1847, 1714, 1642, 1548, 1453, 1410, 1381, 1378, 1342, 1309, 1291, 1267, 1207, 1170, 1074, 1056, 997, 944, 922, 817, 788, 757, 740, 691, 602, 681 cm^-1^;^1^H and ^13^C-NMR (CD_3_COCD_3_, 600 MHz) data (Table 1); HR-ESI-MS *m/z*: 311.1832 ([M+Na]^+^, calc. for C_15_H_28_O_5_ Na, 311.1829).

(*R*^∗^, *E*)-2- (3-(2-hydroxypropan-2-yl) cyclopentylidene) acetic acid (**14**): white amorphous powder: +1.3° (c 0.06, CHCl_3_); UV (CHCl_3_) λ_max (logε): 206.50 (2.72), 217.50 (2.74) and 239.50 (3.14) nm; IR (KBr): ν_max_ = 3302, 3127, 2975, 2956, 2930, 2880, 2667, 2623, 2525, 2168, 2034, 1936, 1906, 1689, 1647, 1466, 1427, 1381, 1310, 1270, 1221, 1175, 1143, 1110, 1088, 1072, 1034, 1002, 965, 921, 867, 826, 770, 734, 694, 618, 572, 543, 499, 475, 445, 416 cm^-1^;^1^H and ^13^C-NMR (CDCl_3_, 600 MHz) data (Table 1); HR-ESI-MS *m/z*: 207.0994 ([M+Na]^+^, calc. for C_10_H_16_O_3_Na_,_ 207.0992).

## Results

### Identification of the Fungus SWUKD3.1410

**FIGURE 2:**
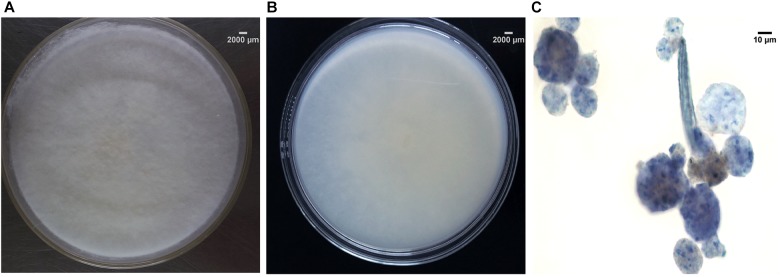
Morphological characteristics and microscopic morphology of SWUKD3.1410. **(A)** Front view of *Umbelopsis dimorpha* SWUKD3.1410 grown on PDA media 3 days; **(B)** Back view of *U. dimorpha* SWUKD3.1410 grown on PDA media 3 days; **(C)** Microscopic structures (multi-spored and single-spored sporangia) of *U. dimorpha* SWUKD3.1410 grown on water-agar media 10 days.

**FIGURE 3:**
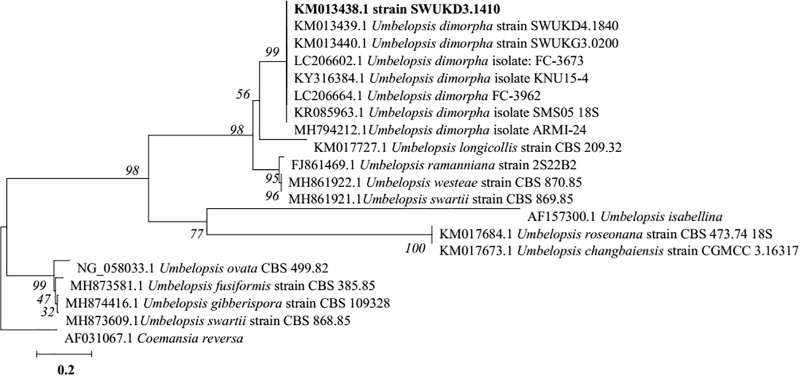
Neighbor-joining tree based on ITS rDNA sequence of the fungus WUKD3.1410 and its closest ITS rDNA matches in the GenBank.

Macroscopic examination of the isolate revealed that the fungus was filamentous, with velvety white mycelia on the surface of colony (Figures 2A,B). Colonies on PDA after 5 days reaching 45 mm diameter at 28°C, 1–4 mm high, flat, or high in the center and low at the edge, often with white sectors, velvety. The colonies on water-agar plates after 10 days tended to be sporangiophores with single-spored sporangia (Figure 2C), which is consistent with the morphological description of *U.* *dimorpha* in the published literature (Mahoney et al., 2004; Wang et al., 2013). The 5.8S rDNA gene sequence of strain SWUKD3.1410 was determined and classified in the genus *Umbelopsis* on the basis of its phylogenetic affiliation. Figure 3 shows phylogeny tree of the isolated fungus based on neighbor-joining analysis compared to other similar fungi stains. Based on molecular taxonomy and morphology investigation of the strain SWUKD3.1410, this fungus was identified as *U. dimorpha*. And the nucleotides sequences were submitted to GenBank and provided a GenBank Accession No. KM013438.

### TLC and HPLC Analysis of Fermented Samples

The acetone extracts of sterilized/non-sterilized *K. angustifolia* plant materials and wheat bran were respectively analyzed by TLC and HPLC methods. As seen in the TLC plates (Supplementary Figure S1A) and the chromatographic profiles of HPLC (Supplementary Figure S1B), the high temperature sterilization process has almost no effect on the chemical components of the *K. angustifolia* plant materials and wheat bran, especially nigranoic acid, the main component of *K. angustifolia*.

**FIGURE 4:**
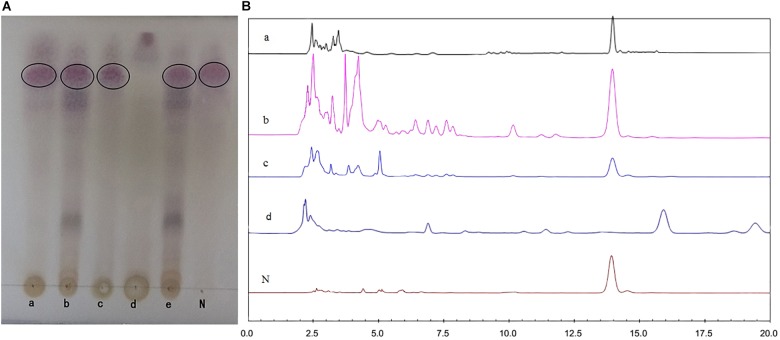
Profiles of chromatograms of extracts from treated different samples. **(A)** Profiles of chromatograms obtained after TLC of the acetone extracts (5 μl of a 1:100 enriched extract) of treated different samples. **(B)** Profiles of HPLC analysis of methanol extracts of treated different samples (5 μl of a 1:100 enriched extract, detection at UV 217 nm). (a) Sterilized *Kadsura angustifolia* control; (b) *U. dimorpha* SWUKD3.1410 grown on *K. angustifolia*; (c) *U. dimorpha* SWUKD3.1410 grown on bran; (d) sterilized wheat bran control; (e) *U. dimorpha* SWUKD3.1410 grown on a mixture of *K. angustifolia* and bran (1:2); N, nigranoic acid (5 μl of 0.1 mg/ml).

The fermentative extracts of *U. dimorpha* SWUKD3.1410 respectively grown on *K. angustifolia* plant materials and wheat bran were analyzed by TLC and HPLC methods. *K. angustifolia* plant materials, wheat bran, nigranoic acid and the culture of *U. dimorpha* SWUKD3.1410 grown on the mixed materials consisting of wheat bran and *K. angustifolia* plant materials (1:2) were respectively served as controls. As seen in the TLC plates (Figure 4A), the chromatographic profile from *K. angustifolia* plant materials was similar to that from *U. dimorpha* SWUKD3.1410 grown on wheat bran while the chromatographic profile from *U. dimorpha* SWUKD3.1410 grown on *K. angustifolia* plant materials was almost identical to that grown on the mixed materials. Conversely, the TLC chromatographic profiles from fermented *K. angustifolia* by *U. dimorpha* SWUKD3.1410 were significantly different from those from *K. angustifolia* plant materials and *U. dimorpha* SWUKD3.1410 grown on wheat bran. Two fermented *K. angustifolia* extracts were revealed to yield more polar compounds than *K. angustifolia* plant materials and single cultures of *U. dimorpha* SWUKD3.1410. The result was proved again by the chromatographic profiles of HPLC analysis (Figure 4B). As for the leached residue of *K. angustifolia*, it was not analyzed because *U. dimorpha* SWUKD3.1410 could not colonize.

### Structural Identification of Compounds From the Fermented *K. angustifolia* Plant Materials

From the acetone extract of the fermented *K. angustifolia* plant materials by *U. dimorpha* SWUKD3.1410, 16 secondary metabolites were obtained. Based on the 1D, 2D-NMR and MS data, their structures were identified as nigranoic acid (**1**) (Dong et al., 2007a)[26], 23α-hydroxynigranoic acid (**2**), abiesatrine J (**3**) (Yang et al., 2010), schisanlactone B (**4**) (Liu et al., 1983), henrischinin A (**5**) (Xue et al., 2011), henrischinin B (**6**) (Xue et al., 2011), lancifodilactone D (**7**) (Li et al., 2004), schirubridilactone D (**8**) (Xiao et al., 2010b), wuweizidilactone D (**9**) (Huang et al., 2007), gomisin D (**10**) (Chen et al., 2005), 6,8-dihydroxy-schensianol A (**11**), schensianol A (**12**) (Zheng et al., 2009), crocinervolide (**13**) (Ortega et al., 1989), (*R*^∗^, *E*)-2- (3-(2-hydroxypropan-2-yl) cyclopentylidene) acetic acid (**14**), 4-hydroxy-3-methoxybenzoic acid (**15**) (Sivakumar et al., 2010), 4-hydroxybenzoic acid (**16**) (Sivakumar et al., 2010), respectively. Among them, compounds **2**, **11**, **14** have not been identified before.

**FIGURE 5:**
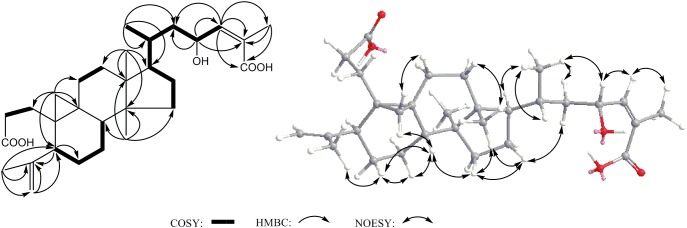
Key COSY, HMBC and NOESY correlations of **2**.

Compound **2** was obtained as acicular crystal and its molecular formula was assigned as C_30_H_46_O_5_ by HR-ESI-MS at *m/z* 509.3228 [M+Na]^+^ (calcd for C_30_H_46_O_5_Na 509.3237) (Supplementary Figure S2). The IR spectrum showed absorptions at 3425, 1695 and 1570 cm^-1^, indicating the presence of hydroxyl, carbonyl and double-bond functionalities. The ^1^H-NMR spectrum showed five methyls [one secondary methyl at δ_H_ 0.92 (d, *J* = 6.2 Hz), two tertiary methyls at δ_H_ 0.90 (s) and 0.96 (s), and two olefinic methyls at δ_H_ 1.71 (s) and δ_H_ 1.66 (s)], an oxymethine at δ_H_ 4.33 (1H, m), one olefinic methine at δ_H_ 6.53 (d, *J* = 7.9 Hz) and one pair of typical cycloartane methylene protons [δ_H_ 0.69 and 0.38 (each 1H, d, *J* = 3.8 Hz)] (Table 1 and Supplementary Figure S3). The ^13^C-NMR and HMQC spectra of **2** resolved 30 carbon signals, corresponding to two carbonyl groups, four double-bonded carbons, four quaternary carbons, five methines (one is oxygenated), 10 methylenes, and five methyls (Table 1 and Supplementary Figures S4, S5). These features closely resembled those of nigranoic acid (**1**) except for the absence of one CH_2_ and the presence of one additional CH-OH group (δ_H_ 4.33 and δ_C_ 64.9), suggesting the OH-substituted nigranoic acid backbone for **2**. This hydroxyl group was located at C-23 because of the ^1^H-^1^H COSY correlation (Figure 5) of H-23 (δ_H_ 4.33, m) with H-24 (δ_H_ 6.53, d, *J* = 7.9 Hz) and HMBC correlations from the oxymethine proton signal at δ_H_ 4.33 (H-23) to C-22 (δ_C_ 43.4), C-24 (δ_C_ 145.3) and C-25 (δ_C_ 126.7), and the olefinic proton at δ_H_ 6.53 (H-24) to C-23 (δ_C_ 64.9) and C-22 (Figure 5 and Supplementary Figure S6). The NOESY correlations of H-23 with C**H**_3_-21 (δ_H_ 0.92, d, *J* = 6.2 Hz) and H-24, and correlations of C**H**_3_-21 with H-17 (δ_H_ 1.54, m), H-20 (δ_H_ 1.68, m), and H_α_-22 (δ_H_ 1.55, m) suggested that the H-23 was β-oriented (Figure 5 and Supplementary Figure S7). This was further confirmed by the absence of NOESY correlation of H-23 with H_α_-22. The NOESY correlation of H-24 with C**H**_3_-26 (δ_H_ 1.71, s), determined the double bond at C-24/C-25 to be *Z* configuration. The similarity of chemical shifts and other observed NOESY correlations suggested other chiral centers possess the same relative stereochemistry as those of nigranoic acid (**1**) (Dong et al., 2007a). Consequently, the structure of **2** (Figure 1) was identified as 23α-hydroxynigranoic acid.

**FIGURE 6:**
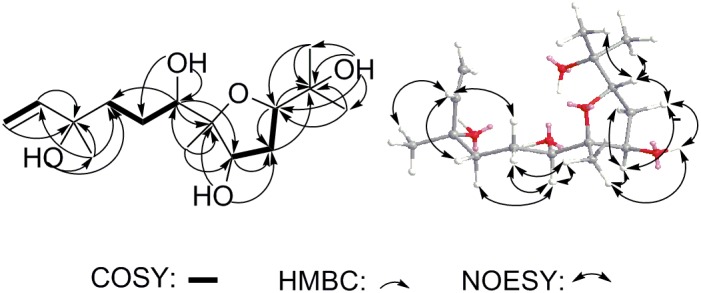
Key COSY, HMBC and NOESY correlations of **11**.

Compound **11** was isolated as colorless oil and assigned to have a molecular formula of C_15_H_28_O_5_ by the HR-ESI-MS at *m/z* 311.1832 ([M+Na]^+^, calcd for C_15_H_28_O_5_Na 311.1829), indicating two indices of hydrogen deficiency (Supplementary Figure S8). Analysis of ^1^H-NMR, ^13^C-NMR, and HMQC data for **11** revealed the presence of two olefinic carbons (including three olefinic protons), along with four methyl groups, three methylene carbons, three oxygenated methine carbons, and three oxygenated quaternary carbons (Table 1 and Supplementary Figures S9–S11). These data accounted for all but four exchangeable protons [δ_H_ 3.57 (s), 3.74 (d, *J* = 4.7 Hz), 3.78 (s) and 4.27 (s)] and required **11** to be monocyclic. Analysis of COSY results (Figure 6 and Supplementary Figure S12) led to the identification of three isolated proton spin-systems corresponding to the C-1-C-2, C-4-C-5-C-6-6-O**H**, and O**H**-8-C-8-C-9-C-10 subunits of structure **11**. The former two units and C-15 (δ_C_ 28.6) were joined at one oxygenated quaternary carbon (C-3, δ_C_ 72.9), as evidenced by the HMBC correlations (Figure 6 and Supplementary Figure S13) of C**H**_3_-15 (δ_H_ 1.21, s) with C-2 (δ_C_ 147.2), C-3 (δ_C_ 72.9), and C-4 (δ_C_ 40.7), and O**H**-3 (δ_H_ 3.78, s) with C-15 and C-4, while the latter two units and C-14 (δ_C_ 17.4) were established to attach to another oxygenated quaternary carbon at C-7 (δ_C_ 88.7) deduced from the HMBC correlations from C**H**_3_-14 (δ_H_ 1.09, s) to C-6 (δ_C_ 78.6), C-7, and C-8 (δ_C_ 73.2), and from O**H**-8 (δ_H_ 3.74, d, *J* = 4.7 Hz) to C-7. Furthermore, the key HMBC correlations from C**H**_3_-12 (δ_H_ 1.18, s) to C-10 (δ_C_ 83.2) and C-11 (δ_C_ 71.4), C**H**_3_-13 (δ_H_ 1.05, s) to C-10 and C-11, and O**H**-11 (δ_H_ 3.57, s) to C-10, C-11, C-12 (δ_C_ 27.4) and C-13 (δ_C_ 26.5) supported a 2-hydroxy propyl unit at C-10. Finally, these data, together with the chemical shifts of C-7 and C-10, indicated that C-7 is connected to C-10 via an oxygen atom, thereby constructing the dihydrofuran moiety of **11**, accounting for the monocyclic structure of **11** required by the molecular formula. Based on the above evidence, the planer structure of **11** was established.

The relative stereochemistry of compound **11** was established by analysis of ^1^H-^1^H coupling constants and NOESY data and by comparison its ^1^H-NMR data with those of the co-existing known metabolite schensianol A (**12**). Specifically, the fact that the oxymethine proton on C-6 exhibited a larger (*J*_H-6,Hα-5_ = 10.2 Hz) coupling constant with H_β_-5 (δ_H_ 1.63, m) but no coupling constant with H_α_-5 (δ_H_ 1.44, m) indicated the hydroxyl group attached to C-6 in a pseudoequatorial orientation. This was also further confirmed by the observed NOESY correlations of H-6 (δ_H_ 3.38, d, *J* = 10.2 Hz) with C**H**_3_-14 (δ_H_ 1.09, s), H_α_-4 (δ_H_ 1.52, m) and H_β_-5 (Figure 6 and Supplementary Figure S14). Additionally, the NOESY correlations of H-8 (δ_H_ 4.32, m) with C**H**_3_-14, H_α_-9 (δ_H_ 2.24, m) and H_β_-9 (δ_H_ 1.79, m), as well as O**H**-8 with H_β_-9 and C**H**_3_-14 indicated a β-orientation for the 8-OH. Accordingly, the structure of **11** was, therefore, deduced to 6,8-dihydroxy-schensianol A, as shown in Figure 1.

**FIGURE 7:**
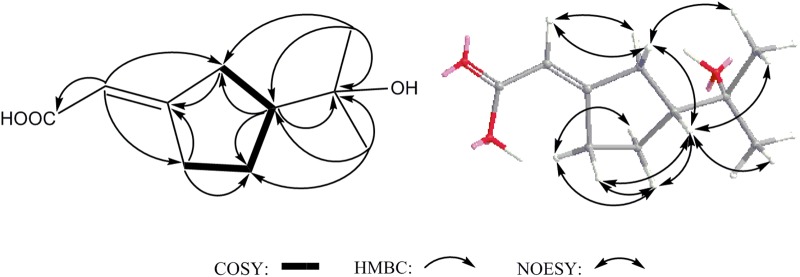
Key COSY, HMBC and NOESY correlations of **14**.

Compound **14** was isolated as white powder and assigned to have a molecular formula of C_10_H_16_O_3_ by the HR-ESI-MS at *m/z* 207.0994 ([M+Na]^+^, calcd 207.0992), indicating three degrees of unsaturation (Supplementary Figure S15). The IR spectrum showed absorptions at 3302, 1689 and 1647 cm^-1^, suggesting the presence of hydroxyl, carbonyl and double-bond functionalities. The ^1^H-NMR spectrum in CD_3_Cl solvent showed the presence of two methyls [δ_H_ 1.21 (s), and δ_H_ 1.22 (s)], and one olefinic methine at δ_H_ 7.04 (s) (Table 1 and Supplementary Figure S16). Analysis of ^13^C-NMR and HSQC data revealed the presence of two methyl groups, three methylenes, one sp^3^ methine, one oxygenated quaternary carbon, two olefinic carbons (one is protonated), and one carbonyl carbon (δ_C_ 170.1) (Table 1 and Supplementary Figures S17, S18). These data accounted for all but two exchangeable protons, and indicated that **14** must contain one carbonyl group and one hydroxyl group. Besides, analysis of COSY results led to the identification of one isolated proton spin-system corresponding to the C-4-C-7 subunit of structure **14** (Figure 7 and Supplementary Figure S19). The HMBC correlations of C**H**_3_-9 (δ_H_ 1.21, s) with C-5 (δ_C_ 44.1) and C-8 (δ_C_ 72.3), and C**H**_3_-10 (δ_H_ 1.22, s) with C-5 and C-8, revealed that C-9 (δ_C_ 27.2), C-10 (δ_C_ 27.4) and the C-5 of C-4-C-7 unit were joined at C-8 (Figure 7 and Supplementary Figure S20). The cross-peaks of H-2 (δ_H_ 7.04, s) with C-4 (δ_C_ 26.5, t) and C-7 (δ_C_ 25.0), H-4 (δ_H_ 2.03, m) with C-3 (δ_C_ 129.9) and C-5, and H-7 (δ_H_ 2.52, m) with C-3 and C-6 (δ_C_ 23.3) led to the direct connection of the quaternary olefinic carbon C-3 to C-4 and C-7, which yielded a cyclopentane ring. The above mentioned HMBC correlations also indicated that the olefinic proton carbon C-2 (δ_C_ 140.5) was connected to the carbonyl C-1 (δ_C_ 170.1). On the basic of these data, the planer structure of **14** was established. The proton at C-5 was determined to be β-oriented by the observed ROESY correlations of H-5 (δ_H_ 1.54, m) with C**H**_3_-9, C**H**_3_-10, H_β_-4 (δ_H_ 2.34, m), H_β_-6 (δ_H_ 1.24, m), and H_β_-7 (δ_H_ 2.17, m) (Figure 7 and Supplementary Figure S21). Additionally, C-2/C-3 double bond was undisputedly determined as having *E*-geometry, judging from the ROESY correlations of H-2 with H_α_-4 (δ_H_ 2.03, m) and H_β_-4. All these evidences established the structure of **14** as (*R*^∗^, *E*)-2- (3-(2-hydroxypropan-2-yl) cyclopentylidene) acetic acid (Figure 1).

### Secondary Metabolites of *U. dimorpha* SWUKD3.1410 Grow on Wheat Bran

From the acetone extract of the endophytic fungus *U. dimorpha* SWUKD3.1410 grow on wheat bran, six secondary metabolites were obtained. Based on the 1D, 2D-NMR and MS data, their structures were identified as nigranoic acid (**1**) (Dong et al., 2007a), schisanlactone B (**4**) (Liu et al., 1983), schisandronic acid (**17**) (Li et al., 2003), 3-methyl ester-nigranoic acid (**18**) (Sy and Geoffrey, 1998), sitostanone (**19**) (Luo et al., 2009), gomisin C (**20**) (Jiang et al., 2005), respectively. Their structures were shown in Figure 1. As far as we know, this is the first study to report nigranoic acid-producing fungi.

## Discussion

Through the long period of co-evolution, some endophytes and their plant hosts have established a special interaction relationship, which can significantly influence the biosynthesis of host metabolism. Previously, the interactions between endophytic fungi and plant hosts have mainly focused on the effects of fungal colonization on secondary metabolism of living host–plant. Almost exclusively, these studies have demonstrated that the fungus–plant interactions relates to the production of secondary metabolites which play a critical role in their interactions, especially for defense, signaling purposes, and regulation of the symbiosis (Schulz and Boyle, 2005). Recently, an alternative approach to study the influence of an endophytic fungus on the host–plant metabolic profiles has been achieved by the analysis of the fungal impacts on the compositions of the host–plant secondary metabolites and their feedback effects on the fungal growth (Tian et al., 2014). Additionally, many studies on microbial transformation have demonstrated that endophytes play an important role in producing novel compounds and modifying their structures and bioactivities (Zeng et al., 2013; Gniłka et al., 2015; Smith et al., 2015). In this paper, in order to obtain highly oxygenated schitriterpenoids/schinortriterpenoids and assess the impact of SWUKD3.1410 on the secondary metabolites of host–plant *K. angustifolia*, *U. dimorpha* SWUKD3.1410 was respectively grown on dead *K. angustifolia* plant materials and wheat bran, and their corresponding fermented products were analyzed.

When cultivated in bran, the unexpected results were obtained that *U. dimorpha* SWUKD3.1410 also produced nigranoic acid (**1**), which was the major active component of the host plant *K. angustifolia* (Huang et al., 2015). Compound **1** has been revealed to possess various bioactivities, including cytotoxicity toward hela and leukemia cell lines, and inhibition of HIV reverse transcriptase and polymerase expression (Sun et al., 1996; Shi et al., 2015). Here, relatively high amounts of **1** were found in the wheat bran cultures of *U. dimorpha* SWUKD3.1410, producing at levels of over 0.37% of the crude extracts. Besides **1**, three additional triterpenes (**4**, **17**, **18**), one sterol sitostanone (**19**) along with one dibenzocyclooctadiene lignan gomisin C (**20**) were found in the cultures of *U. dimorpha* SWUKD3.1410 as well, which were similar to the characteristic components of host plant *K. angustifolia* (Gao et al., 2008; Sun et al., 2011). Taken together with the reported chemical components of wheat bran (Posner, 2009; Zhu et al., 2013, 2015; Prinsen et al., 2014; Geng et al., 2015), we considered that these metabolites (**1**, **4**, and **17**–**20**) should be synthesized by *U. dimorpha* SWUKD3.1410 rather than from the medium. Additionally, the fact that the *U. dimorpha* SWUKD3.1410 yielded the same and similar compounds as its host–plant *K. angustifolia* (Sun et al., 2011) demonstrated that partial or complete biosynthesis pathways of *K. angustifolia* can be attributed to the endophytic fungus *U. dimorpha* SWUKD3.1410. The ability of endophytic fungi to produce host–plant secondary metabolites has been postulated as a horizontal gene transfer between endophytic fungi and host plant for a long time. That is, fungus may obtain the biosynthetic gene as plasmid or extra chromosomal element from its host plant or *vice versa* (Sachin et al., 2013). However, it has not yet been confirmed although there has been one report on horizontal gene transfer between plant and its symbiotic bacteria (Taghavi et al., 2005). Additionally, some researches have demonstrated that the pathway of endophytic fungi biosynthesizing the same plant metabolite was completely different from that of plant hosts (Boemke and Tudzynski, 2009). For example, the *hyp*-1 gene responsible for the biosynthesizing hypericin by *Thielavia subthermophila* was found to be absent in its host plant *Hypericum perforatum* (Kusari et al., 2009). Occasionally, it has been strongly suggested that the interactions between plant hosts and endophytes contribute to the co-production of bioactive secondary metabolites (Heinig et al., 2013). In light of these, we assumed that the biosynthesis of nigranoic acid both by *K. angustifolia* and *U. dimorpha* SWUKD3.1410 may be related to its ecological adaptability to environmental conditions, and such includes the ready exchange of genetic information that enables the endophytic fungus to mimic the chemical environment of its host and, ultimately, to acquire a lot of evolutionary superiorities because no other *U. dimorpha* strains have been detected to synthesize schitriterpene-type metabolites (Tan and Zou, 2001; Wang et al., 2017). Further comparative study on the genomes between *U. dimorpha* SWUKD3.1410 and other strains may shed light on the molecular mechanisms which trigger same and/or similar chemical reactions of Schisandraceae plant with *U. dimorpha* SWUKD3.1410.

From *K. angustifolia* plant materials fermented by a symbiont endophytic fungus, *U. dimorpha* SWUKD3.1410, 16 secondary metabolites (**1**–**16**) were isolated and identified, including three new metabolites (**2**, **11**, and **14**). Of them, metabolites **1**–**6** belong to schitriterpenoids while **7**–**9** are schinortriterpenoids. Specifically, abiesatrine J (**3**) was a rare C-24/C-25 double bond isomer of nigranoic acid (**1**) and was only found in two plants, *Abies georgei* Orr (Yang et al., 2010) and *Hopea odorata* Roxb (Satiraphan et al., 2015). Henrischinins A-B (**5**–**6**) were previously isolated from *Schisandra henryi* (Xue et al., 2011) and *S. chinensis* (Song et al., 2013), and reported to have moderate to weak activity against HSV-2 and adenovirus (Boemke and Tudzynski, 2009) and HL-60 cell lines (Xue et al., 2011). Biogenetically, the rare 3-oxo-2-oxabicyclo[3,2,1]octane moiety of **5**–**6** probably arose from schisanlactone B (**4**) after a Michael addition as proposed by Xia and coworkers (Xia et al., 2014). As a key intermediate, **4** was repeatedly found in *S. propinqua* (Chen et al., 2001), *Schisandra henryi* (Chen et al., 2003), and *K. heteroclita* (Wang et al., 2006) and reported to have moderate cytotoxic activity against Leukemia cells and HL-60 cells *in vitro* (Heinig et al., 2013). As for schinortriterpenoids **7**–**9**, what the most worth mentioning was that they were obtained many times from the genus *Schisandra* but never from genus *Kadsura* so far (Huang et al., 2008; Lei et al., 2009; Xiao et al., 2010a; He et al., 2012). From the viewpoint of chemical structures, schitriterpenoids **2**–**6** and schinortriterpenoids **7**–**9** were considered to probably arise from nigranoic acid (**1**), the main component of *K. angustifolia* plant, because they have never been detected in our previous chemical investigation on the same samplings (Sun et al., 2011), although we found that the *U. dimorpha* SWUKD3.1410 yielded the same and similar compounds as its host–plant *K. angustifolia*. Furthermore, the plausible biosynthetic pathways of these products have been well-proposed in the previous reviews on triterpenoids from the Schisandraceae family (Xiao et al., 2008; Shi et al., 2015). Finally, it should be noted that our study will be interesting if **7**–**9** were transformed products of nigranoic acid (**1**), because this is the first discovery of highly oxygenated nortriterpenoids by microbial technology. Compared with our reported biotransformation on the substrate nigranoic acid by the same fungus (Wang et al., 2017), the present investigation to detect and isolate highly oxygenated schitriterpenoids/schinortriterpenoids from *K. angustifolia* fermented by its associated endophytic fungus, *U. dimorpha* SWUKD3.1410 was successful, indicating perhaps the necessity of specific host plant environment for microbial conversion.

With regard to compound **10**, it was elucidated as a known lignan, gomisin D, which possessed a unique structure with an additional 11-membered ring resulting from a cyclization of a tigloyl moiety with a phenolic hydroxyl group and no double bond in the cyclooctadiene ring. Previously, **10** was isolated from *S. chinensis* (Mocan et al., 2016) and *S. grandiflora* (Poornima et al., 2016) and suggested that not its amount but rather its chemical structure was responsible for the highest scavenging activity in the DPPH cuvette assay and the best ability to inhibit tyrosine nitration in 14 tested lignins (Mocan et al., 2016). In another paper, gomisin D (**10**) showed potent inhibition of the AChE activity with the IC_50_ value as 7.84 ± 0.62 μM, implying that it may be the active component in the plants that is useful in treatment of Alzheimer’s disease (Hung et al., 2007). In addition, this investigation also led to the discovery of one monoterpene (**14**), one trinorsesquiterpenoid (**13**), two sesquiterpenoids (**11**–**12**), and two simple aroma compounds (**15**–**16**). To our knowledge, only a few sesquiterpenoids and monoterpenoids have been obtained from several species of *Kadsura*, such as *K.* *heteroclita* (Roxb.) (Li and Luo, 2002), *K. longipedunculata* (Pu et al., 2008), *K. interior* (Dong et al., 2013). However, the terpenes (**11**–**14**) found in the fermented *K. angustifolia* materials were completely different from them. From the literature report, these terpenoids and phenolics are essential to the plant’s healthy growth because they may provide the plant defense against fungi, bacteria, helminths, and insects in its natural environment (Chadwick et al., 2013).

As hypothesized by Demain: if a fungus can produce metabolites *in vitro* they must have a function in nature. The multienzyme reaction cascade required for the synthesis of secondary metabolites would not be retained by fungi without some beneficial effect for survival (Demain, 1980). Accumulating evidence has demonstrated that endophytic colonization may improve the host’s ecological adaptability under the environment stress by producing antimicrobial metabolites against phytopathogens and predators (Tan and Zou, 2001; Schulz and Boyle, 2005). As previously found for pathogenic fungi, the plants whose roots are usually colonized by endophytes often grew faster than non-infected ones (Pegg and Ayres, 1984). The phenomenon was supposed to be due to the synthesis of plant hormones and other growth-promoting substances by fungi (Petrini, 1991; Tudzynski, 1997; Tudzynski and Sharon, 2002). Recently, several reports have described the influence of fungal endophytes on the secondary metabolites of host–plant. It was notably the case that the induction of phenols was observed after infection with an endophytic fungus *Colletotrichum tropicale* from the Clavicipitaceae family, which was found to discourage leaf-cutting ants (Estrada et al., 2013). In another case, *Paraconiothyrium variabile*, a leaf endophytic fungus isolated from the leaves of *Cephalotaxus harringtonia*, was unraveled to induce a special delycosylation of the host plant flavonoids for its own growth in the host–plant (Tian et al., 2014). Taken together, we assumed that metabolites **1**–**20** found here in the endophytic fungus *U. dimorpha* SWUKD3.1410 may play a fundamental role within the host, and (or) have ecological significance, even if this required more research to confirm.

In a conventional view, it is widely considered that the quality and quantity of crude drugs originated from medicinal plants is highly correlated to the genetic background, ecological habitats, and soil nutrients of the concerned plants. Recently, it is gradually recognized that endophytic microbes have served a very important function in enhancing the quality and quantity of medicinal plants through a specific fungus–host interaction (Jia et al., 2016). Endophytic fungi have been found to have fundamental impacts on the biogeographic distribution of host plant species. Especially for those plants that have to rely on certain endophytic fungi to provide nutrients to complete their life history, endophytic fungi are more likely to be an important limiting factor (Jia et al., 2016). For several medicinal plants, it has been indicated that they are active due to their microbial endophytes (Li et al., 2012; Miller et al., 2012). Additionally, among the various so-called “plant metabolites,” especially those exclusive to their host plants, many have been detected in cultivated endophytic fungi (Kusari et al., 2012), as shown in our study. Occasionally, certain plant metabolites can’t be synthesized without the involvement of fungi (Kusari et al., 2011a,b). On the other hand, the colonization of endophytic fungi is not random due to the chemotaxis which is specific chemicals produced by the host plants (Carroll and Petrini, 1983). Generally, the distribution of certain endophytic fungal populations was only restricted to particular genetic background of a species and particular host plant species (or families). Under certain special conditions, the distribution of endophytic fungi from the same regions is highly similar, while species and their population structure of endophytic fungi even in the same host plant species from different regions usually have very low similarity (Jiang et al., 2010). In light of these, we assumed that genuine medicinal materials with the highest quality and best effects to a certain disease had a special relationship with endophytic fungi of medicinal plants. Additionally, it should be noted that the cultivation of endophytes without their host may lead to the loss of the desired compound synthesis, since the plant host can change or influence the metabolic pattern of secondary metabolites in endophytic fungi (Ludwig-Müller, 2015). Thus, more understanding on the beneficial symbiosis can help to enhance the synthesis and accumulation of bioactive metabolites of the host medicinal plants for higher quality of crude drugs.

## Author Contributions

DQ and LW conceived and designed the experiments. DQ, LW, and JW performed the experiments. DQ and JD analyzed the data. MH, HS, XY, and XD contributed reagents, materials, and analysis tools. DQ and LW wrote the paper.

## Conflict of Interest Statement

The authors declare that the research was conducted in the absence of any commercial or financial relationships that could be construed as a potential conflict of interest.
