# Supplementary material for: Effects of an Endophytic Fungus Umbelopsis dimorpha on the Secondary Metabolites of Host–Plant Kadsura angustifolia
Source: Front Microbiol. 2018 Nov 22;9:2845. doi: 10.3389/fmicb.2018.02845 (PMC6262151; doi:10.3389/fmicb.2018.02845)
Supplement: Supplementary file 1 [file Data_Sheet_1.docx]

**Effects of an endophytic fungus *Umbelopsis dimorpha* on the secondary metabolites of host-plant *Kadsura angustifolia***

**Dan Qin ^1, &^, Ling Wang ^1, &^, Meijun Han ^1^, Junqi Wang ^1^, Hongchuan Song ^2^, Xiao Yan ^1^, Xiaoxiang Duan ^1^ & Jinyan Dong ^1,^ ***

^1^ Key Laboratory of Eco-environments in Three Gorges Reservoir Region, Ministry of Education, School of Life Sciences, Southwest University, Chongqing 400715, People's Republic of China

^2^ School of Energy and Environment Science, Solar Energy Research Institute, Yunnan Normal University, Kunming 650092, People's Republic of China

**^&^**These authors contributed equally to this work and should be considered co-first authors

***Correspondence author**: Dr. Jinyan Dong. Address: Key Laboratory of Eco-environments in Three Gorges Reservoir Region (Ministry of Education), School of Life Sciences, Southwest University, Chongqing, 400715, China

Fax: 023-68252400. E-mail: [donjyaa@swu.edu.cn](mailto:donjyaa@swu.edu.cn)

**List of Supplementary Materials**

Fig. S1. Profiles of chromatograms of extracts from treated different samples. (A) Profiles of chromatograms obtained after TLC of the acetone extracts of treated different samples (5 *µ*l of a 1:100 enriched extract). (B) Profiles of HPLC analysis of methanol extracts of treated different samples (5 µl of a 1:100 enriched extract, detection at UV 217 nm). a: non-sterilized *K. angustifolia*; b: sterilized *K. angustifolia*; c: non-sterilized wheat bran; d: sterilized wheat bran; N: nigranoic acid (5 µl of 0.1mg/ml).


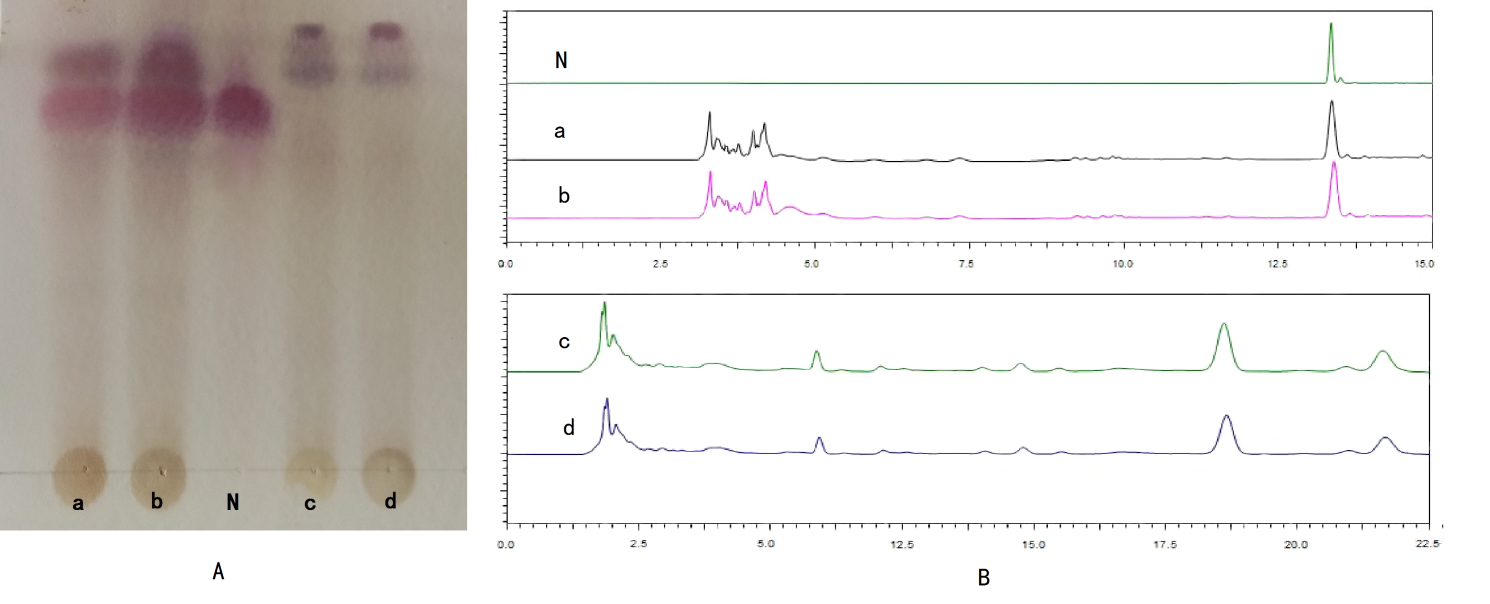


Fig. S2. HR-ESI-MS spectra of new compound **2** (CD_3_OD).

Fig. S3. ^1^H-NMR spectra of new compound **2** (CD_3_OD).

Fig. S4. ^13^C-NMR spectra of new compound **2** (CD_3_OD).

Fig. S5. HMQC spectra of new compound **2** (CD_3_OD).

Fig. S6. HMBC spectra of new compound **2** (CD_3_OD).

Fig. S7. NOESY spectra of new compound **2** (CD_3_OD).

Fig. S8. HR-ESI-MS spectra of new compound **11** (CD_3_COCD_3_).

Fig. S9. ^1^H-NMR spectra of new compound **11** (CD_3_COCD_3_).

Fig. S10. ^13^C-NMR spectra of new compound **11** (CD_3_COCD_3_).

Fig. S11. HMQC spectra of new compound **11** (CD_3_COCD_3_).

Fig. S12. ^1^H-^1^HCOSY spectra of new compound **11** (CD_3_COCD_3_).

Fig. S13. HMBC spectra of new compound **11** (CD_3_COCD_3_).

Fig. S14. NOESY spectra of new compound **11** (CD_3_COCD_3_).

Fig. S15. HR-ESI-MS spectra of new compound **14** (CDCl_3_).

Fig. S16. ^1^H-NMR spectra of new compound **14** (CDCl_3_).

Fig. S17. ^13^C-NMR spectra of new compound **14** (CDCl_3_).

Fig. S18. HMQC spectra of new compound **14** (CDCl_3_).

Fig. S19. ^1^H-^1^HCOSY spectra of new compound **14** (CDCl_3_).

Fig. S20. HMBC spectra of new compound **14** (CDCl_3_).

Fig. S21. ROESY spectra of new compound **14** (CDCl_3_).


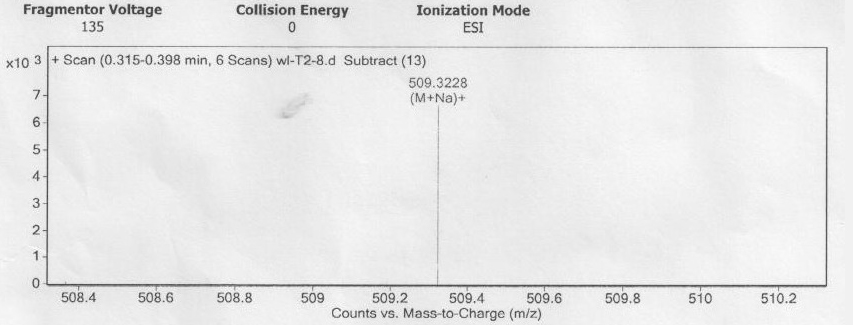


Fig. S2. HR-ESI-MS spectra of new compound **2** (CD_3_OD).


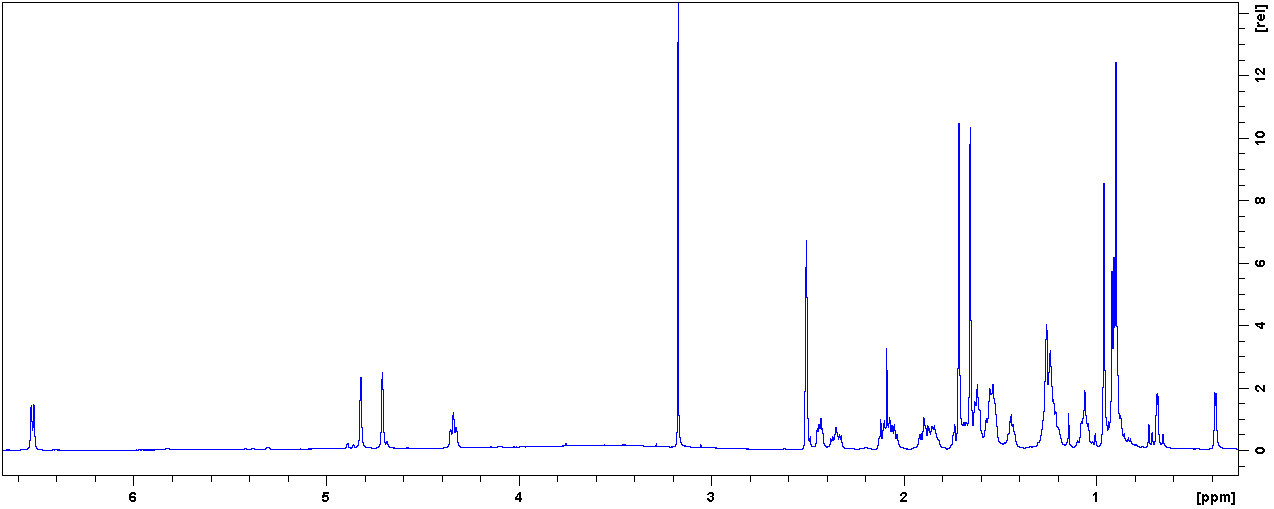


Fig. S3. ^1^H-NMR spectra of new compound **2** (CD_3_OD).


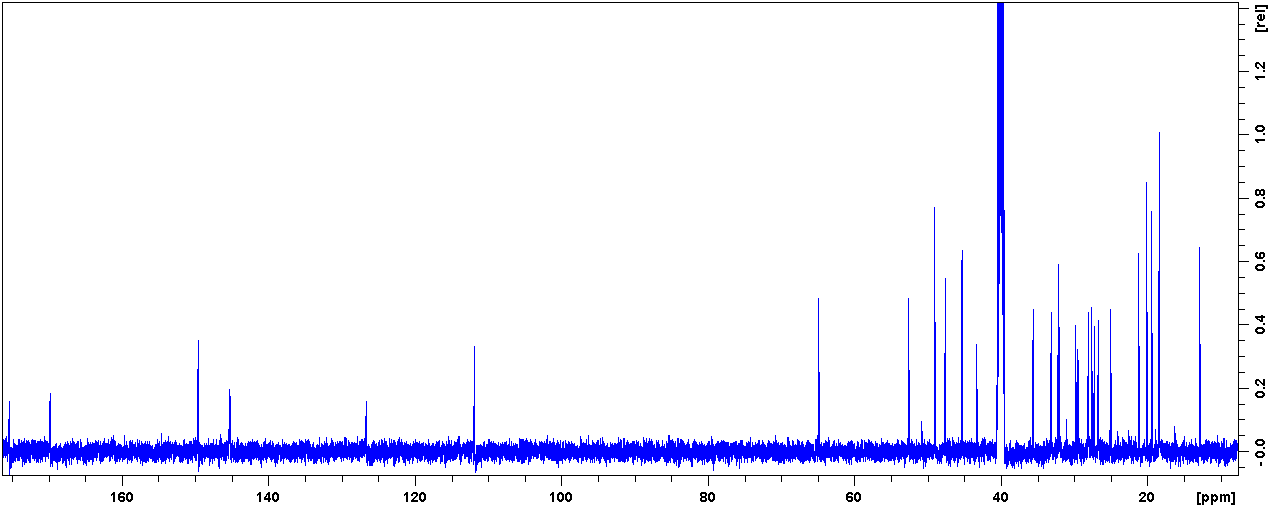


Fig. S4. ^13^C-NMR spectra of new compound **2** (CD_3_OD).


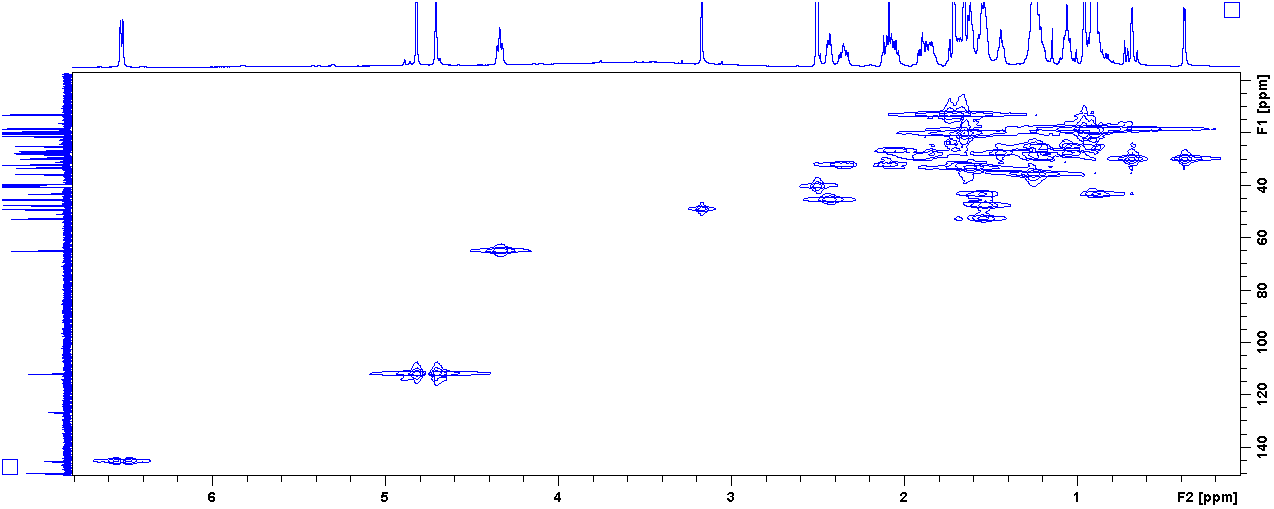


Fig. S5. HMQC spectra of new compound **2** (CD_3_OD).


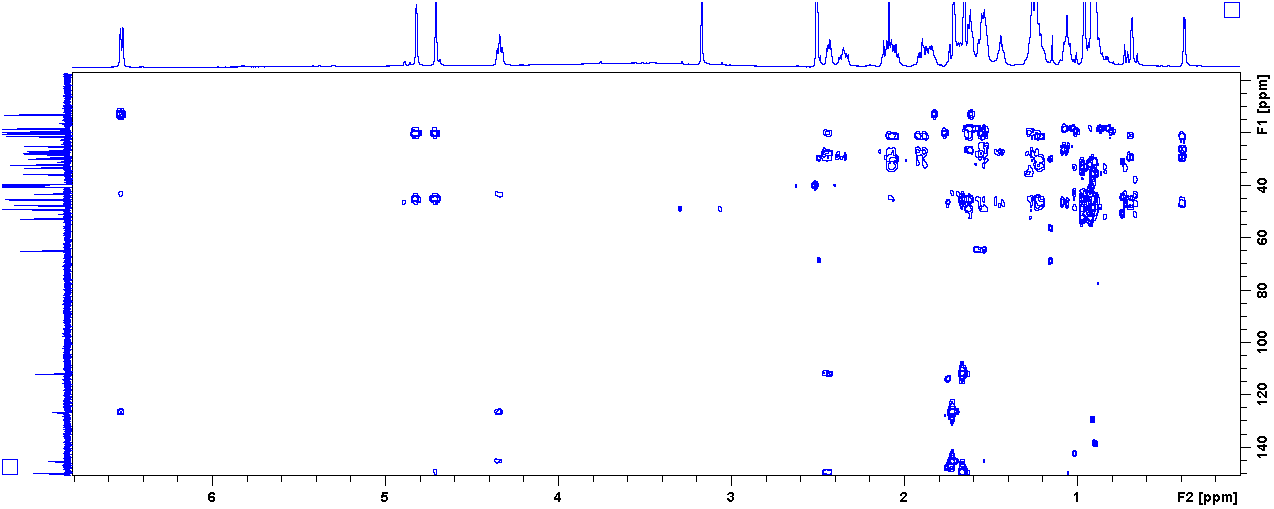


Fig. S6. HMBC spectra of new compound **2** (CD_3_OD).


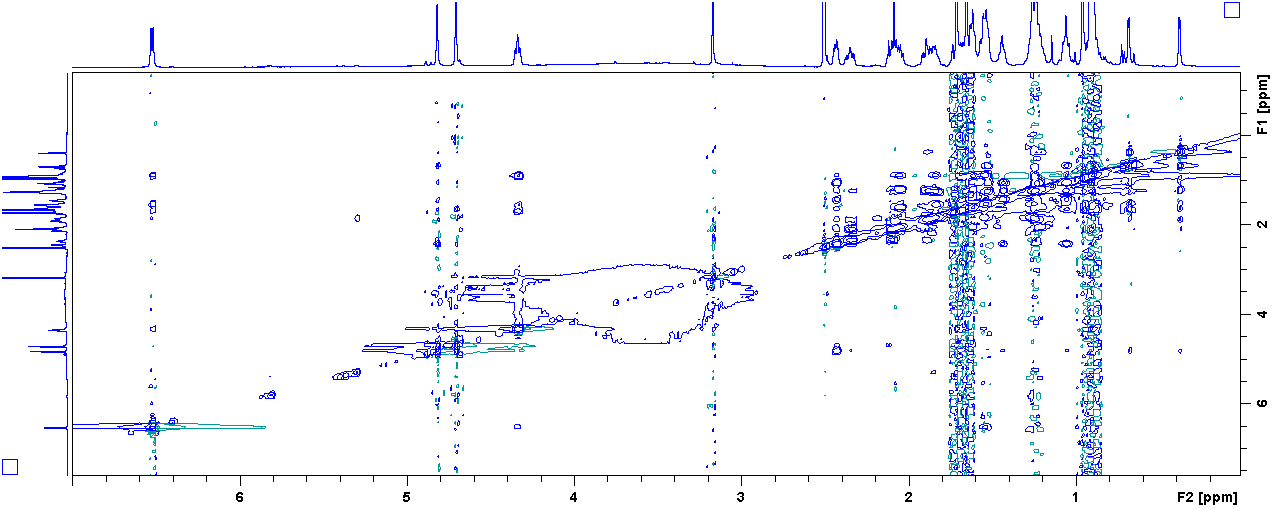


Fig. S7. NOESY spectra of new compound **2** (CD_3_OD).


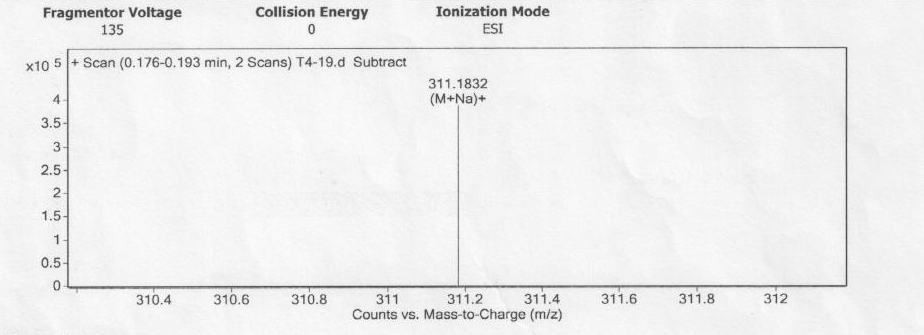


Fig. S8. HR-ESI-MS spectra of new compound **11** (CD_3_COCD_3_).


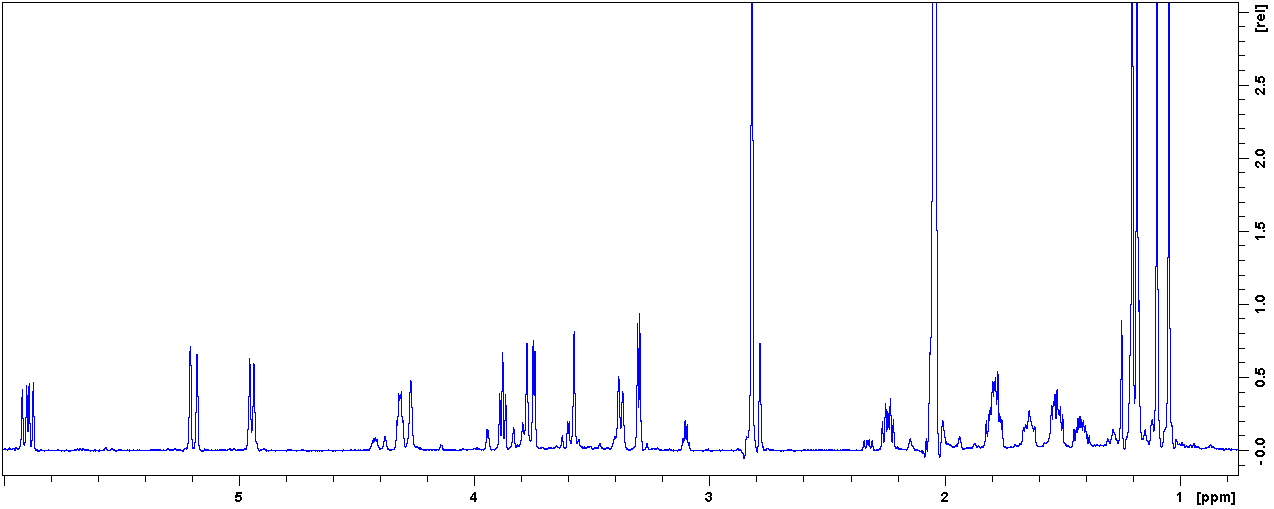


Fig. S9. ^1^H-NMR spectra of new compound **11** (CD_3_COCD_3_).


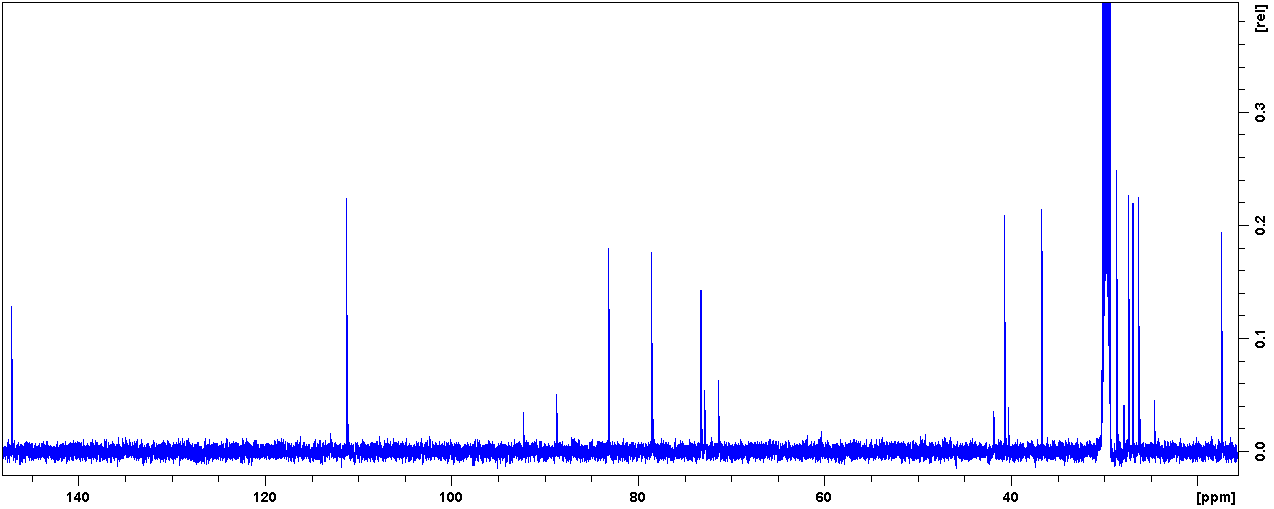


Fig. S10. ^13^C-NMR spectra of new compound **11** (CD_3_COCD_3_).


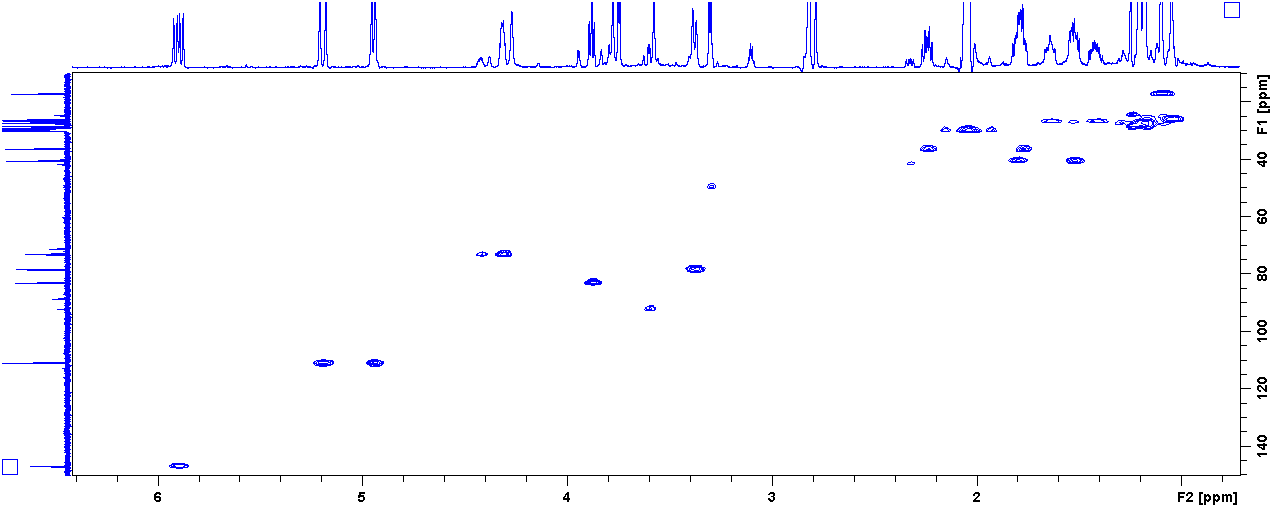


Fig. S11. HMQC spectra of new compound **11** (CD_3_COCD_3_).


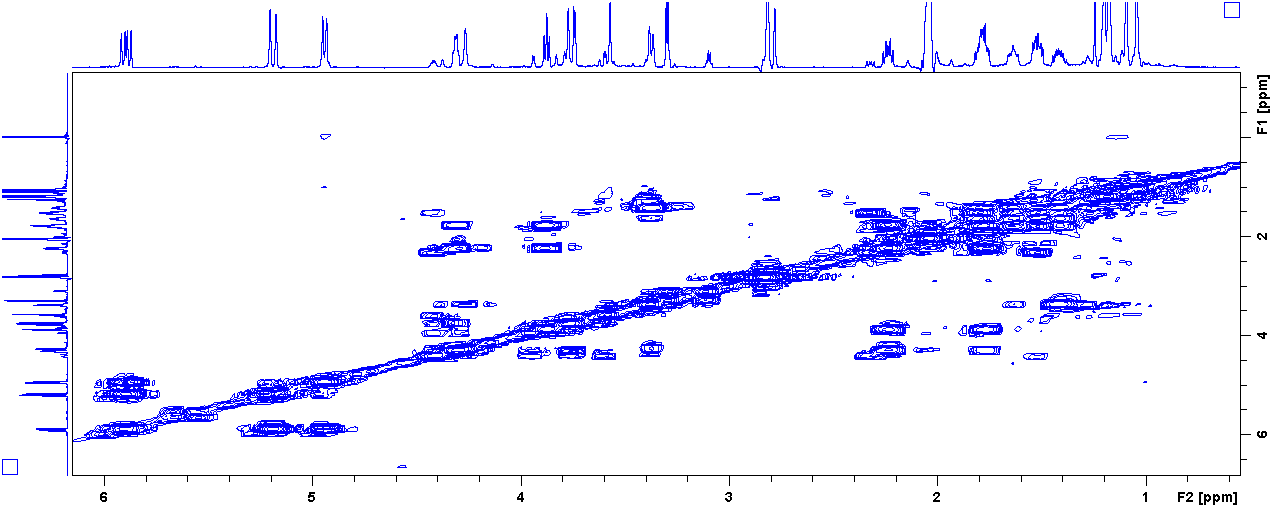


Fig. S12. ^1^H -^1^H COSY spectra of new compound **11** (CD_3_COCD_3_).


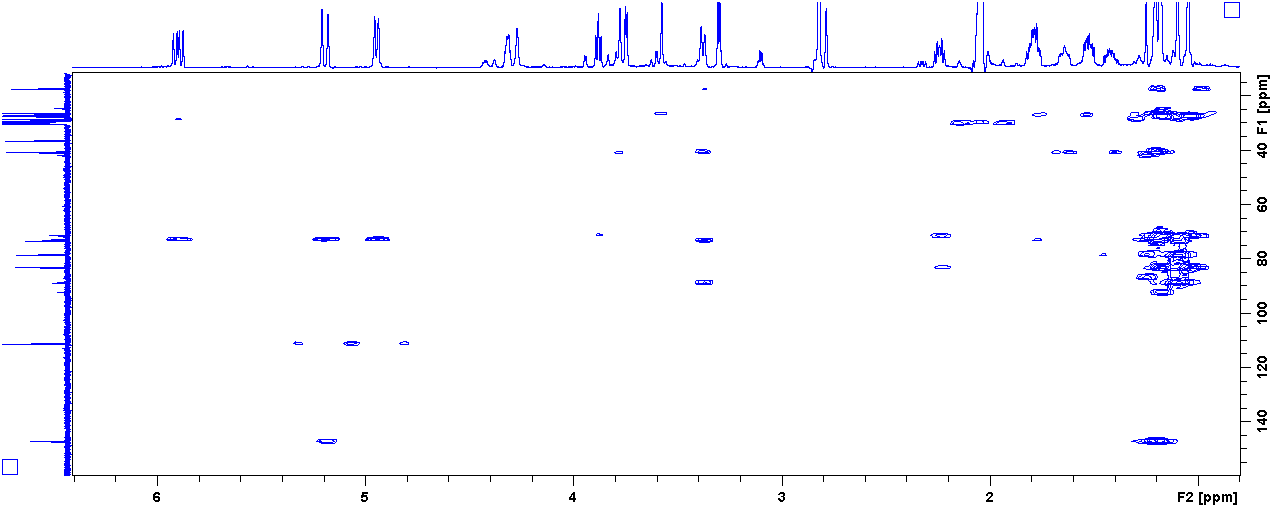


Fig. S13. HMBC spectra of new compound **11** (CD_3_COCD_3_).


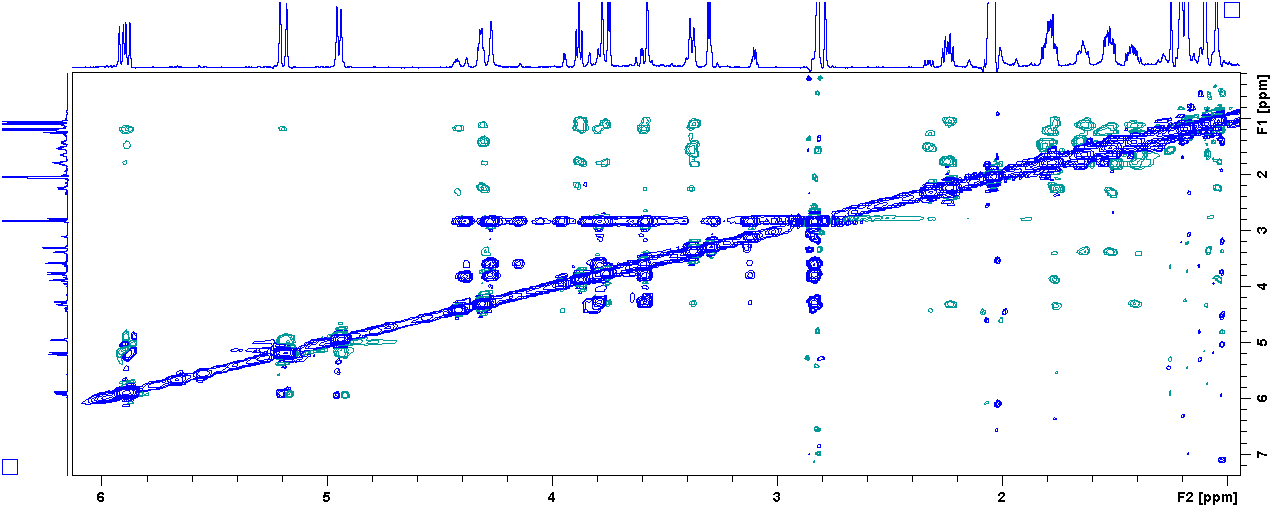


Fig. S14. NOESY spectra of new compound **11** (CD_3_COCD_3_).


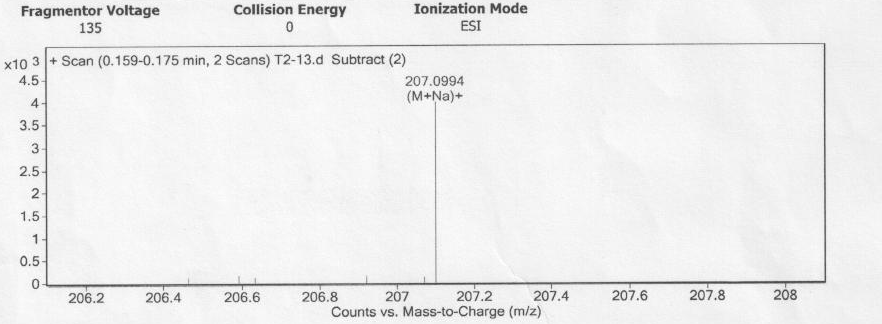


Fig. S15. HR-ESI-MS spectra of new compound **14** (CDCl_3_).


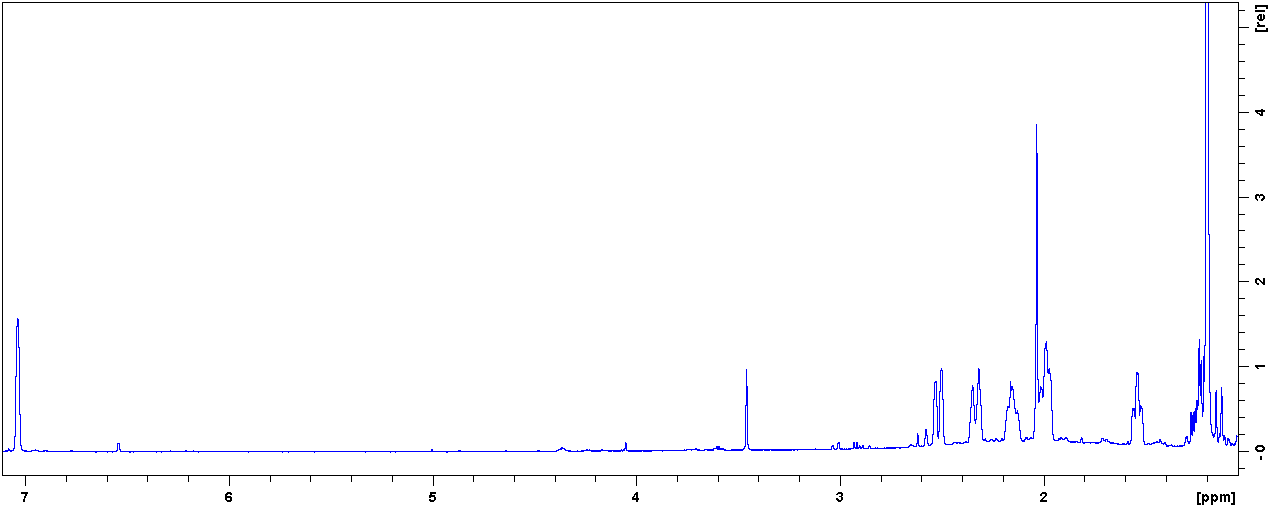


Fig. S16. The ^1^H-NMR spectra of new compound **14** (CDCl_3_).


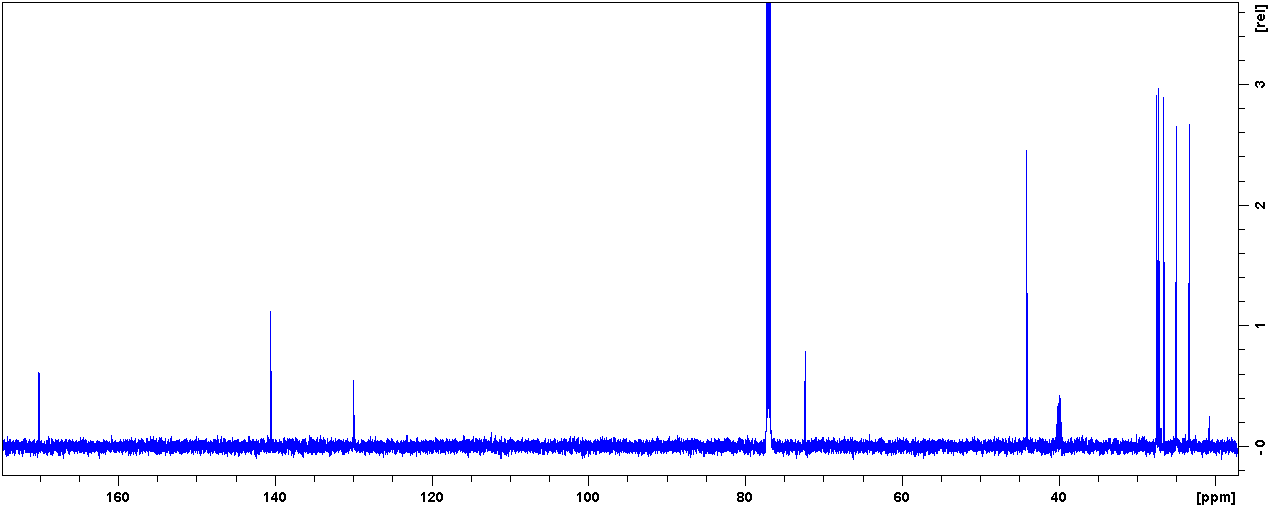


Fig. S17. The ^13^C-NMR spectra of new compound **14** (CDCl_3_).


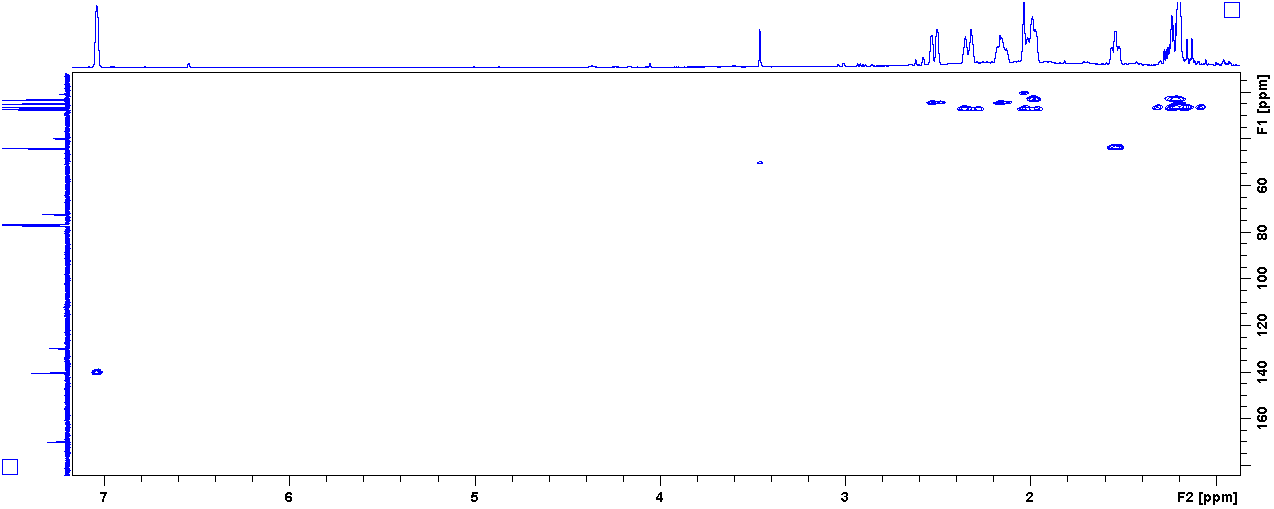


Fig. S18. HMQC spectra of new compound **14** (CDCl_3_).


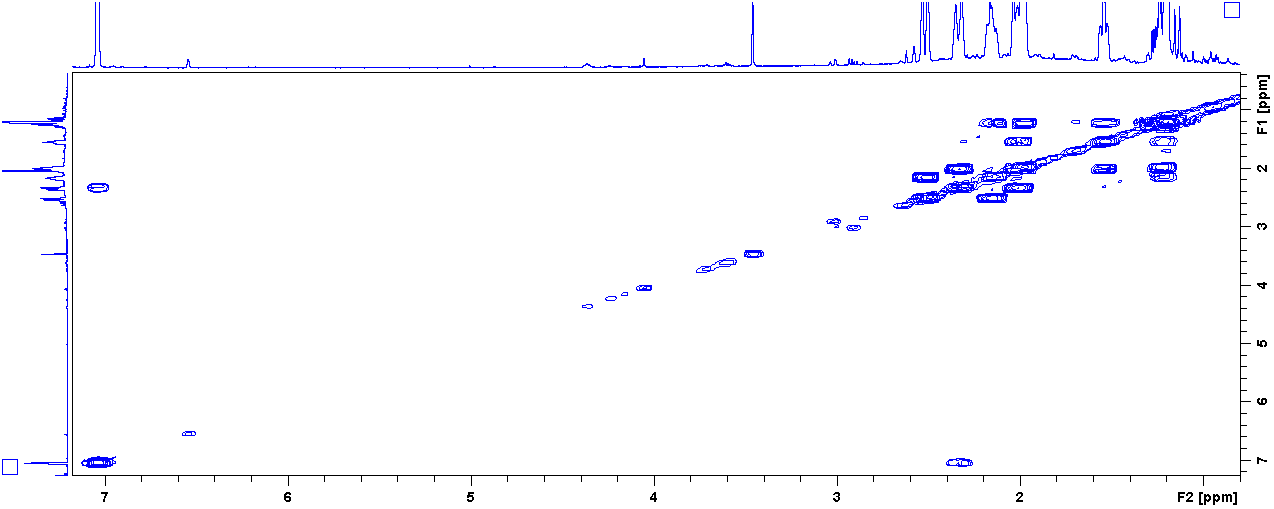


Fig. S19. ^1^H -^1^H COSY spectra of new compound **14** (CDCl_3_).


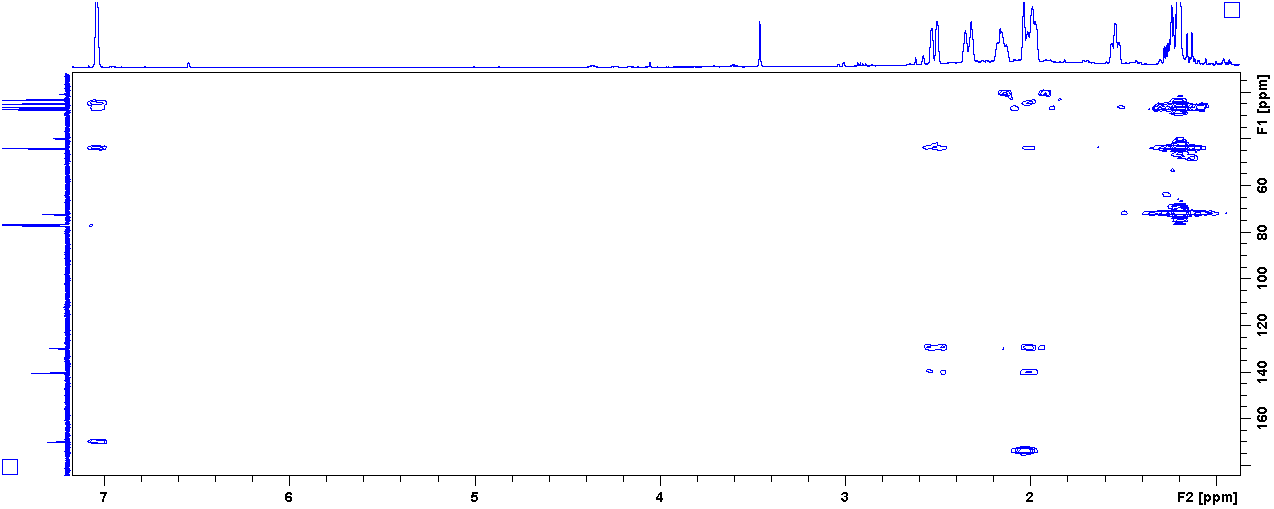


Fig. S20. HMBC spectra of new compound **14** (CDCl_3_).


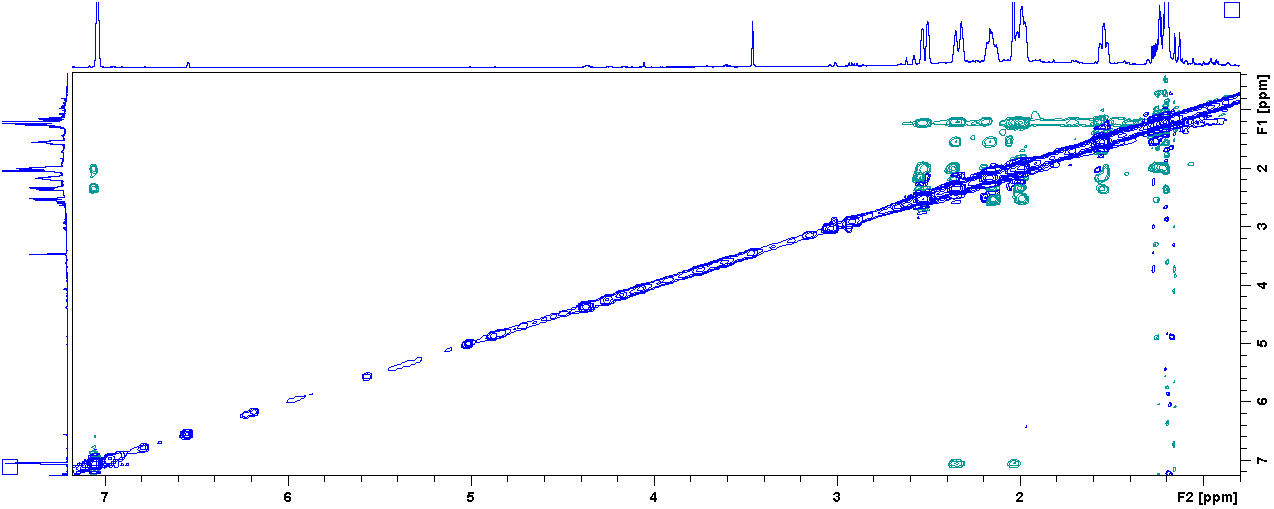


Fig. S21. ROESY spectra of new compound **14** (CDCl_3_).
